# New Metrics for Comparison of Taxonomies Reveal Striking Discrepancies among Species Delimitation Methods in *Madascincus* Lizards

**DOI:** 10.1371/journal.pone.0068242

**Published:** 2013-07-12

**Authors:** Aurélien Miralles, Miguel Vences

**Affiliations:** 1 Division of Evolutionary Biology, Zoological Institute, Technical University of Braunschweig, Braunschweig, Germany; 2 CNRS-UMR5175 CEFE, Centre d’Ecologie Fonctionelle et Evolutive, Montpellier, France; Consiglio Nazionale delle Ricerche (CNR), Italy

## Abstract

Delimiting and describing species is fundamental to numerous biological disciplines such as evolution, macroecology, and conservation. Delimiting species as independent evolutionary lineages may and often does yield different outcomes depending on the species criteria applied, but methods should be chosen that minimize the inference of objectively erroneous species limits. Several protocols exploit single-gene or multi-gene coalescence statistics, assignment tests or other rationales related to nuclear DNA (nDNA) allele sharing to automatically delimit species. We apply seven different species delimitation protocols to a taxonomically confusing group of Malagasy lizards (*Madascincus*), and compare the resulting taxonomies with two newly developed metrics: the Taxonomic index of congruence C*_tax_* which quantifies the congruence between two taxonomies, and the Relative taxonomic resolving power index R*_tax_* which quantifies the potential of an approach to capture a high number of species boundaries. The protocols differed in the total number of species proposed, between 9 and 34, and were also highly incongruent in placing species boundaries. The Generalized Mixed Yule-Coalescent approach captured the highest number of potential species boundaries but many of these were clearly contradicted by extensive nDNA admixture between sympatric mitochondrial DNA (mtDNA) haplotype lineages. Delimiting species as phenotypically diagnosable mtDNA clades failed to detect two cryptic species that are unambiguous due to a lack of nDNA gene flow despite sympatry. We also consider the high number of species boundaries and their placement by multi-gene Bayesian species delimitation as poorly reliable whereas the Bayesian assignment test approach provided a species delimitation highly congruent with integrative taxonomic practice. The present study illustrates the trade-off in taxonomy between reliability (favored by conservative approaches) and resolving power (favored by inflationist approaches). Quantifying excessive splitting is more difficult than quantifying excessive lumping, suggesting a priority for conservative taxonomies in which errors are more liable to be detected and corrected by subsequent studies.

## Introduction

Species are the fundamental unit for a wide array of biological studies and applied fields such as conservation planning [1]. The rise of new genomic and bioinformatic tools led to claims that the time is ripe for a comprehensive mission to explore and document millions of yet undescribed species in the next decades, as a basic tool to understand and reverse the biodiversity crisis on Earth [2]. For the first time in history, such a mission is technically possible yet it implies reconsidering and partly re-orienting current work protocols of taxonomy. “Fast-track taxonomy” is in reach [3]–[6] and requires discussing how to accelerate taxonomic description while maintaining and even increasing the quality of species hypotheses. Species must be delimited as objectively and rigorously as possible but the great majority of the known species on Earth have been – and are still – discovered and described using comparative morphology only, an approach fundamentally unchanged for centuries and yet woefully understudied from conceptual and methodological perspectives [7]. Conceptual advances in taxonomy such as the understanding of species as independent population-level lineages [8], [9], and a renewed interest in species delimitation [10], [11], have led to an integrative perspective in taxonomic practice [12]–[14]. This implies that evidence from different species criteria (SC), oriented on patterns or processes of lineage splitting [15], can be used to support species hypotheses. Taxonomic practice, however, is only rarely based on explicit approaches to the testing of species hypotheses, and only recently have software implementations of species delimitation methods been developed (e.g., [16]–[22]).

Combining molecular and morphological evidence in taxonomy has been advocated early on [12], [23], [24], and provides excellent perspectives to identify genealogical lineages and assess their evolutionary independence. Despite the development of rigorous approaches [23], these are rarely applied in practice, and most taxonomic studies that combine molecular and morphological data eventually use to some degree expert opinion to evaluate which of the monophyletic units in a gene genealogy has the “requisite” morphological characteristics to be recognized as species (e.g., [25]). The common discordance between species trees and gene trees, caused by incomplete lineage sorting, hybridization, gene duplication, reticulated evolution, or recombination [26] is rarely taken into account in taxonomic practice despite some exemplar studies (e.g., [27]–[30]). Multilocus molecular data hold the promise to draw hypotheses of independent evolutionary lineages, i.e., species, with much higher confidence than pure morphological or DNA barcoding approaches. Yet they also imply the risk of severe taxonomic oversplitting, given that also intraspecific units can be distinguished at a fine scale on the basis of allele frequencies and often even by fixed alleles of fast evolving markers such as microsatellites. Such fine scale units can be flagged as evolutionary significant units, ESUs, or management units for conservation, MUs [31] but in most cases do not correspond to species under any SC. Simulations can be used to test the relative performance of species delimitation approaches to identify diverging lineages at different points in evolutionary time (e.g., [17]). However, only comparing the performance of these approaches in empirical case studies can align them with current taxonomic practice and probe for possible failure caused by the usually incomplete and biased data of such real-world data sets.

In line with the identification of species delimitation as a Renaissance subject [10] explicit approaches are now emerging that exploit single-gene or multi-gene coalescence statistics, assignment tests or other rationales related to nuclear DNA allele sharing to automatically delimit species [16], [17], [21], [32]–[34]. These methods are increasingly used in case studies on a variety of organisms [35]–[40]. This has partly generated intense controversy among taxonomists [41]–[43] confirming that the routine application of any of these methods in taxonomy should be preceded by comparative assessments of their performance in a variety of organisms groups. Because different species criteria will identify independent lineages at different stages of the lineage splitting process [9] it is obvious that species delimitation approaches can differ in their outcome. On the other hand errors in species delimitation in some cases can be objectively detected, drastic examples being those where different stages or sexes of one interbreeding population are assigned to two species, or where reproductively incompatible sympatric lineages are assigned to a single species. Avoidance of such objective errors should be a main criterion to prefer certain species delimitation approaches over others, and this requires comparatively determining their relative error rates.

Such a comparison of species-level taxonomies is not straightforward. While various metrics exist to compare the topology of phylogenetic trees (e.g., [44], [45]) methods to compare classifications surprisingly are still in their infancy. Typically, the performance of species delimitation methods and effect of applying alternative species criteria is quantified by the number of different species recognized in each case (e.g. [16], [35], [43]) but without considering similarity or difference in the content of each of the units considered as species, i.e., are the species recognized in two taxonomies equally delimited? Recently, Sauer and Hausdorf [43] used the Rand index to compare species delimitation results, a metric that takes into account the assignment of specimens to clusters. While useful with balanced sampling sizes, this index will be impacted more strongly by assignment differences in clusters represented by a large number of specimens than by differences in specimens-poor clusters. In the present study we propose two metrics that can be used for taxonomy comparisons without being biased by the number of specimens per species, and apply them to species delimitation in a group of skinks from Madagascar, the genus *Madascincus*.

Within the monophyletic group of Malagasy scincines, the genus *Madascincus* with currently 10 species has a particularly confusing taxonomy as (i) several nominal taxa likely represent complexes of at least two undescribed species (eg. within *M. polleni* or *M. igneocaudatus*, [46], [47]) whereas on the contrary (ii) the taxonomic validity of several species is uncertain as their morphological distinctiveness has not been tested with adequate sample sizes (e.g. *M. ankodabensis*, *M. minutus, M. intermedius*). Threat status and distribution of reptiles have been used for defining conservation priorities in Madagascar, one of the most diverse and most imperiled biodiversity hotspots [48]. Such assessments typically use species as unit for analysis and therefore heavily depend on the quality of species hypotheses. *Madascincus* serve as a good model for the difficulties in achieving a reliable taxonomy because sampling success by pitfall trapping of the various species can strongly differ between sites thus leading to a biased sampling, and morphological homoplasy appears to be common [47]. On the other hand, the sympatric occurrence of different lineages within this group also offers the advantage to confirm or reject species hypotheses by assessing admixture or lack thereof in a natural setting.

Here we selected, of the plethora of methods for species-delimitation proposed over the last years, seven distinct methods that (i) do not depend on a-priori assignment of specimens to clusters or species, (ii) are being applied in the practice in recent publications, especially to squamates or to taxa from Madagascar, and (iii) can be implemented without a specific a-priori sampling design (e.g., across contact zones). These methods are based on explicit protocols which greatly minimize the need of subjective interpretations or taxonomic expertise for the studied group. Two are exclusively based on nDNA and mtDNA, respectively, two combine nDNA and mtDNA, and one combines mtDNA and morphological data. We apply these methods to the *Madascincus* data set and compare the results based on the two new metrics developed herein, the Taxonomic index of congruence C*_tax_* and the Relative taxonomic resolving power index (R*_tax_*). Rather than further refining any of these methods we reproduce their originally proposed and/or routinely used implementation. We then evaluate their relative performance and error rate, by defining as main yardstick an integrative taxonomy work protocol that makes use of all available evidence for species boundaries.

## Materials and Methods

### Ethics Statement

No experiments were conducted using living animals. All field researches, collecting of specimens, including in situ euthanasia of specimens were approved by the Madagascan Ministère de l’Environnement, des Eaux et des Forets (Direction des Eaux et Forets, DEF) under the following permits: 156-MEF/SG/DGEF/DADF/SCB dated 12 December2002; 238MINENVEF/SG/DGEF/DPB/SCBLF dated 14 November 2003; 238MINENV.EF/SG/DGEF/DPB/SCBLF/RECH dated 22 December 2004; 272MINENV.EF/SG/DGEF/DPB/SCBLF/RECH dated 8 November 2005; 298MINENV.EF/SG/DGEF/DPB/SCBLF/RECH dated 22 December 2006; 036/08 MEEFT/SG/DGEF/DSAP/SSE dated 30January; 2008;26/09/MEEFT/SG/DGEF/DSAP/SLRSE dated3 February 2009; 48/09/MEEFT/SG/DGEF/DSAP/SSE dated9 March 2009; 188/09/MEEFT/SG/DGEF/DSAP/SSE dated16 September 2009; 195/09/MEEFT/SG/DGEF/DSAP/SSE dated 28 September 2009; 314/10/MEF/SG/DGF/DCB.SAP/SCB dated 4 November 2010. Export of specimens was approved by the DEF under permits: 063C-EA02/MG03, dated 26 February 2003; 094C-EA03/MG04, dated 1 March 2004; 103C-EA03/MG05, dated 15 March 2005; E1400/06, dated 1 June 2006; 055N-EA03/MG10, dated 25 March 2010. Import of species protected by CITES into Germany was approved by the German authorities (Bundesamt fur Naturschutz). Voucher specimens were euthanized using approved methods (e.g. anaesthesia with ketamine, followed by ketamine overdosis and 95% ethanol fixation) that do not require approval by an ethics committee after consultation of the animal welfare officer of TU Braunschweig.

### Taxonomic Framework

The definition of the genus *Madascincus* herein follows previous molecular work [49]–[52], encompassing all species of an exclusively four-legged lineage that is sister to the legless genus *Paracontias*. Throughout the manuscript we use species names (scientific binomina) largely following current taxonomy (see File S1). In this scheme, our sampling contains seven of eight nominal species in *Madascincus* and only misses *M. macrolepis* which almost certainly is closely related to *M. nanus* due to numerous morphological similarities. We emphasize that species names herein merely serve to unambiguously refer to certain clusters of specimens under current taxonomy. They do not imply an assumption of an optimal species hypotheses, and do not represent the outcome of any of these.

### Samples, Specimens and Morphology

For the molecular analyses, 157 tissue samples of *Madascincus* were collected across Madagascar between 2001 and 2010. A piece of tissue was removed and stored in 96% ethanol, and representative voucher specimens fixed in 5% formalin or 95% ethanol and stored in 70% ethanol. We examined morphology of a total of 168 preserved specimens, not all studied also with molecular methods but including most type specimens in the genus. Specimens came from the Muséum National d’Histoire Naturelle, Paris (MNHN), Museo Regionale di Scienze Naturali, Torino (MRSN), National History Museum, London (NHM), Forschungsinstitut und Naturmuseum Senckenberg, Frankfurt am Main (SMF), Université d'Antananarivo, Département de Biologie Animale (UADBA), and Zoologische Staatssammlung München (ZSM). FGZC, FG/MV, MV, MgF refer to Frank Glaw, Miguel Vences and Madagascar Frontiers field numbers. Lists of all voucher specimens used for morphological and molecular study as well as geographical coordinates of collecting localities are included in File S2. Measurements of specimens were recorded to the nearest 0.1 mm using a dial caliper. Meristic, mensural and qualitative characters examined here are those routinely used in the taxonomy of Scincidae, such as scale counts, presence or absence of homologous scale fusions, or color pattern (details in File S3). Scale nomenclature, scale counts, and measurements follow previous studies [47], [53].

### Molecular Data and Phylogenetic Analysis

We collected DNA sequence data for two fragments of two mitochondrial (mtDNA) genes, NADH-dehydrogenase subunit 1 (ND1) with adjacent tRNAs (tRNAMet, tRNAGln and tRNAIle genes) and 16S rRNA (16S), and for four protein-coding nuclear genes (nDNA), brain-derived neurotrophic factor (BDNF), recombination activating gene 2 (RAG2), oocyte maturation factor (CMOS) and phosducin (PDC). Standard polymerase chain reactions were performed in a final volume of 12.5 μl containing 0.3 μl each of 10 pmol primer, 0.25 μl of total dNTP 10 mM (Promega), 0.1 μl of 5 U/ml GoTaq, and 2.5 μl of GoTaq Reaction Buffer (Promega). Primers and PCR conditions are given in File S4 and followed Crottini et al. [51]. The successfully amplified products were purified using the ExoSAP-IT purification kit according to the manufacturer’s instruction. Purified PCR templates were sequenced using dye-labeled dideoxy terminator cycle sequencing on an ABI 3130 automated DNA sequencer. The data matrix is 99 % complete, missing only 7 sequences from a possible total of 954. Sequences were aligned using the ClustalW algorithm and subsequently refined manually in BioEdit 7.0 [54]. We used GBLOCKS [55] with stringent settings (no gaps allowed) to determine and exclude uncertain positions in the alignment, but also calculated trees with all positions included which showed similar topology and support. A total of 909 newly determined sequences were deposited in GenBank under accession numbers JQ007903–JQ008811 (see File S5).

Guide trees play a critical role in several species delimitation methods (e.g. [23], [38]). We used a main guide tree reconstructed from the concatenated mtDNA data set (16S and ND1), but we also inferred trees from the unphased concatenated nDNA (BDNF, RAG2, CMOS, PDC) and from the combined nDNA+mtDNA, following concatenation and species tree approaches, in order to ensure that their respective topologies were congruent with the mtDNA tree. We follow Li and Lecointre [56] in considering the recovery of congruent topologies from independent data sets (in this case, unlinked loci) as one of the most relevant criteria to assess clade reliability.

Phylogenetic analysis by Bayesian inference (BI) was carried out using MrBayes 3.1.2 [57]. Models of evolution were determined for each gene by AIC in MrModeltest 2.3 [58]: GTR+G+I for ND1 and 16S rRNA, GTR+G for CMOS, K80+I for BDNF, K80+G for PDC, and HKY for RAG2. Additional analyses were carried out defining partitions by codon (see File S6). For each analysis we performed one run of 20 million generations (started on random trees) and four incrementally heated Markov chains (using default heating values) each, sampling the Markov chains at intervals of 1000 generations. The convergence of the Markov chains was checked with the Tracer v1.5 [59] and mixing of chains was assessed with AWTY [60]. The first 10 million generations were conservatively discarded and 10000 trees were retained post burn-in and summed to generate a 50%-majority rule consensus tree. As hierarchical out-groups, we used *Amphiglossus meva* and *Paracontias fasika*, the latter belonging to the sister genus of *Madascincus* [51], [61].

Coalescence-based species trees were inferred using *BEAST 1.7.4 [62]. This approach might outperform concatenated data sets in the reconstruction of phylogenetic relationships (e.g., [63]) but depends on a-priori assignment of specimens to species and its use in species delimitation can lead to circular reasoning. We therefore used it on assignments of specimens based on analysis with STRUCTURE (see Files S6, S7) and with the main purpose to confirm the topology of the concatenated analysis. Each analysis consisted of combining six runs with MCMC chains set to 500 million generations each, and with settings for each partition as in the MrBayes analyses. Parameter files were examined in Tracer 1.5 to ascertain convergence and adequate effective sample sizes. Species tree files were combined in Tree Annotator 1.7.2 with a conservative burn-in of 50%.

### Species Delimitation Protocols

In this paper, we selected for comparison a total of seven approaches to species delimitation that combine various lines of evidence or are based on explicit models of evolution. Four of these – Bayesian Assignment Test (BAT), Haploweb method (HW), Bayesian Species Delimitation (BSD) and Generalized Mixed Yule Coalescent approach (GMYC) – have been relatively recently proposed and at least partly automate the species delimitation process by specific software, and we applied them using prior settings or interpretations of results in order to minimize species number overestimation and thereby maximizing the reliability of species boundaries [13]. From the plethora of available approaches [10], [11] we manually applied one – Wiens and Penkrot protocol (WP) – that partly reflects taxonomic practice and that is applicable to data typically gathered in taxonomic revisionary work, i.e., with limited sampling of specimens and populations: the method of Wiens and Penkrot [23] based on mitochondrial gene trees only. We further implemented two approaches – Mitochondrial Tree Morphological Character congruence (MTMC) and Integrative Taxonomy (ITAX) – that we consider represent the current state-of-the-art and that combine different data and different lines of evidence in an integrative way [13]. We apply all these approaches following the originally described methodology as it is not our goal to develop them further but to compare their outcomes. All methods are in the following shortly described, with more details on the work procedures in Supporting information file.

Especially in the context of DNA barcoding, species delimitation is often based on pure distance methods in which a differentiation of specimens or populations above a certain threshold of genetic divergence is considered as species criterion (e.g., [22], [64], [65]). A similar rationale has long been applied to allozyme distances (e.g. [66]) and relies on the fact that genetic differentiation correlates with reproductive isolation [67]. Indeed such methods can yield species limits concordant with those inferred by other methods [14]. Still, we here reiterate our previously expressed opinion [13], [68], that distance data alone should only be used to provide a preliminary identification of candidate species but not routinely as sole evidence in species delimitation (see also [69]), and therefore we have not included such approaches.

We follow Padial et al. [13] considering in the context of taxonomy as alpha error the probability of false positives (wrongly delimiting a group of specimens as distinct species), and as beta error the probability of false negatives (failure to detect and to delimit an independent evolutionary lineage, i.e., a species). Outgroups used for phylogenetic inference were not included in species delimitation analyses.

i We define as *Mitochondrial Tree – Morphological Character Congruence* (MTMC) the formalization of a method that according to our experience at present represents the most common practice in those zootaxonomic studies combining evidence from DNA sequences and morphological data. It recognises as species those morphologically diagnosable units that are revealed by a mtDNA tree, i.e., it follows a morphological approach informed by a molecular tree. An assumption is that the mtDNA tree is not strongly influenced by introgression events between species; we suggest that this can be excluded by assessing topological congruence of the mtDNA tree with a tree derived from nuclear gene data [5], preferably based on several nuclear markers. Fixed and unambiguous morphological character states, such as presence or absence (for qualitative characters), non-overlapping values (for meristic or mensural characters), unambiguously differentiated color pattern, or distinct modes of reproduction, represent strong evidence for reduced or absence of gene flow [1]. MTMC is then an iterative process of comparing morphological data with the mtDNA tree seeking for the least inclusive monophyletic group in the molecular tree that is characterized by at least one unambiguously diagnostic morphological character.
ii The *Integrative Taxonomic approach (ITAX)* follows the principle that as many lines of evidence as available should be combined to delimit species [12], [13], [24]. Observations from many different fields of research might provide conclusive evidence for the independence of lineages and thus their identity as different species, but several of them might not be applicable to particular cases, such as those relying on particular geographical settings. A non-exhaustive list of species delimitation criteria to be integrated in this approach includes: (a) sympatric occurrence without admixture as revealed by consistent differences, even if weak, in morphological or molecular characters at the same geographic location; (b) strong differences in a behavioral, morphological or genetic character known to mediate premating isolation; (c) unviability or infertility of hybrids; (d) lack of gene flow across a geographical hybrid zone [70]; (e) congruent diagnostic differences between sister lineages in various unlinked morphological character (respecting the need for minimum sample sizes of specimens and populations); (f) absence of haplotype sharing in several unlinked nuclear loci (again taking sample sizes into account); (g) a combination of criteria e-f; (h) if minimum sample sizes are not met, a diagnostic difference in at least one morphological character which in the respective taxonomic group is known to be highly stable within species, and where the divergent state is not easily attributable to a malformation. Several other lines of evidence, such as the method of Good and Wake [71] to identify genetically isolated groups by rejecting isolation-by-distance, or point or chromosome mutations known to lead to reproductive incompatibility could be added to the list of criteria to be used in ITAX.

We here propose a formalization of this approach which uses the mtDNA guide tree after assessing that no indication for massive mtDNA introgression exists (as in MTMC above). Emphasis is on mtDNA (as available for instance from DNA barcoding studies) not as a means to accurately reconstruct the phylogeny but to define a starting hypothesis of clustering of specimens. Species delimitation is based on seeking the least inclusive monophyletic group in the mtDNA tree which fulfils at least one of the criteria listed above (for a detailed work protocol, see File S8). ITAX therefore minimizes the alpha-error by only taking into account the most unambiguous species evidence provided by a variety of approaches (mostly approaches of integration by congruence [13]), and attempts to keep the beta-error low by seeking evidence from as many different approaches as possible.

iii The protocol developed by Wiens and Penkrot in 2002 *(WP)* delimit species on the basis of nonrecombining molecular phylogenetic data [23]. This approach is designed to test for the status of a focal species, and relies on comparing the phylogenetic pattern of specimens assigned to this species relative to other, closely related species. It follows a flow chart leading to alternative species-level decisions, assuming gene flow (and thus conspecificity) among populations whose specimens do not form exclusive lineages. We applied this method to the *Madascincus* mtDNA gene tree by testing separately the status of each of the nominal species recognized above in the *taxonomic framework* section. For a detailed work protocol, see File S9.
iv The *Bayesian Assignment Test (BAT)* is based on the assumption that speciation starts when populations become genetically separated through a significant reduction in gene flow. It assumes that genetic patterns generated by population-level processes operating within diverging lineages contain the signal of speciation even when divergence is too recent to have generated phylogenetic patterns of independent evolution, such as exclusive monophyly for multiple nuclear loci, whereas reciprocal monophyly in mitochondrial DNA gene trees is supposed to occur relatively rapidly after speciation due to a reduced effective population size. The aim of this method is to combine population genetic and genealogical patterns across multiple loci and to recognize species according to concordance observed between mtDNA clades and patterns of nuclear population structure [33], [71].

Our implementation of this approach strictly followed Weisrock et al. [33]. We assessed population structure based on the four nuclear loci in STRUCTURE v2.2 [72], [73]. Analyses were performed under models assuming a range of 2 to 18 populations (K). This analysis assigns individuals probabilistically to clusters based on their multilocus genotype. We estimated posterior distribution based on two million MCMC generations of which 50% were discarded as burnin. We used a model that considers the possibility of mixed population ancestry and of correlated allele frequencies among populations due to migration or shared ancestry [73]. We estimated the log (ln) probability of the data (X) for each K [ln Pr(X|K)] and calculated ΔK [74] with Structure Harvester [75]. Plots were visualized with Microsoft Excel. We based species delimitation on the correspondence between nuclear clusters and clades in the mtDNA gene tree (BI tree of concatenated ND1 and 16S sequences). We identified the optimal clustering solution based on the posterior probability of the analysis and a plateau of the ln Pr(X|K) probabilities for replicated analyses and compared this solution with other solutions of higher K values, and repeated the analyses for subsets of taxa, i.e., separately for the *melanopleura* clade and the *polleni-stumpffi-arenicola* clade which both comprise morphologically similar taxa and were highly supported as monophyletic groups in all analyses. In a few cases, discrepancies were observed between clusters and mt DNA tree topology (“non-monophyletic distribution” of a cluster across the tree; cf. Results), which we assessed on a case-by-case basis.

v The *Haploweb (HW)* method views species as *fields for recombination* [76], [77], characterized by mutual allelic exclusivity. It uses haplotype networks with additional connections between haplotypes found co-occurring in heterozygous specimens (haplowebs) to delineate species boundaries [32], and therefore can only be used with nuclear DNA in diploid or polyploid taxa.

We used the PHASE algorithm [78] implemented in DnaSP v5 [79] to infer haplotypes from the nuclear DNA sequences. Haplotype networks were reconstructed using statistical parsimony [80], as implemented in the program TCS v1.21 [81] with a connection limit of 95%. Networks were imported in Adobe Illustrator to add colors, connections between co-occurring haplotypes and the number of specimens in which haplotypes were found co-occurring [32]. Following a conservative approach, we based species delimitation on a majority consensus of the results inferred from all four markers: two populations were considered as distinct species if at least three out of four markers congruently reconstructed them as distinct fields for recombination.

vi *Bayesian Species Delimitation (BSD)* is based on coalescence theory. In the absence of recent admixture between species, bipartitions of specimens in gene trees that are shared across loci can potentially be used to infer the presence of two or more species. However, genealogies for individual loci are often poorly resolved and that ancestral lineage sorting, hybridization, and other population genetic processes can lead to discordant gene trees. BSD generates posterior probabilities of species assignments taking into account the uncertainties due to unknown gene trees and the ancestral coalescent process [17].

We applied BSD using Bayesian Phylogenetics and Phylogeography software (BPP v.2.1, [17], [82]) with the phased data set for the four nuclear loci. BSD accommodates the species phylogeny as well as lineage sorting due to ancestral polymorphism, assuming no admixture following speciation. For practical reasons (see File S10), analyses were applied separately to four different species groups of *Madascincus* undisputably representing heterospecific clades: the *M. polleni* group, the *M. melanopleura* group, the *M. igneocaudatus* group and the *M. mouroundavae* group. For each of these four groups, ten, nine, six and two sublineages were respectively assumed as species to be tested. Delimitation analysis was not applied within *M. nanus* because the few samples available for this highly distinct taxon came all from the same locality and presented no significant variability (occurrence of single haplotypes for the mtDNA marker and for three of the four nDNA markers). User-specified guide trees were derived from BI of the concatenated mitochondrial sequences. Separate rjMCMC analyses initiated with different starting seeds, each with 100,000 generations (each fifth sampled) and a burn-in of 10,000 produced consistent results. Ensuring adequate rjMCMC mixing involves specifying a reversible jump algorithm to achieve dimension matching between species delimitation models with different numbers of parameters, and we used algorithm 0 with the fine-tuning parameter *ε* = 15. Additionally, the program was run a few times with *ε = *10 or 20 for the same algorithm, or using algorithm 1 with default fine-tuning parameters (*α = 2* and *m* = 1), to ensure stability among runs [17]. Each species delimitation model was assigned equal prior probability. The prior distributions of the ancestral population size (θ) and root age (τ_o_) can affect the posterior probability for models, with large values for θ and small values for τ_o_ favoring conservative models containing fewer species [17]. We evaluated the influence of these priors by considering three different combinations as in Leaché and Fujita [38], assigning both priors a gamma G(α, β) distribution, with a prior mean = α/β and prior variance = α/β^2^. Each analysis was run at least twice to confirm consistency between runs. Following a conservative approach, only speciation events simultaneously supported by probabilities superior or equal to 0.99 for all three combinations of priors were considered for species delimitation. For a detailed work protocol, see File S10.

vii The *Generalized Mixed Yule-coalescent approach (GMYC)* is based on a statistical model testing for the predicted change in branching rates at the species boundary of a single-locus phylogenetic tree (typically based on short mtDNA fragments generated by DNA barcode studies and not usually used with nuclear DNA sequences). The overall aim of the procedure is to classify the observed branching time intervals defined by the nodes in a chronogram to differentiate between inter-specific (“diversification”) and intra-specific (“coalescent”) processes of lineage branching [16].

We calculated a chronogram derived from the concatenated mitochondrial data set using BEAST [62] with model settings, generations and output evaluation as in species tree estimation with *BEAST (see above). This ultrametric tree was analyzed with the GMYC package (designed to be used in conjunction with APE, in R language) to determine the sharp increase in branching rate that presumably marks the transition from between-species to within-species rate of lineage branching. Both the single threshold version [16], [83], and the multiple threshold extension [35] were applied and compared using a log likelihood ratio test as implemented in the GMYC package. A lineage-through-time plot as produced by the software was visually evaluated for changes in branching rate.

### New Statistical Tools for Assessment and Comparison of Taxonomies

**Figure 1:**
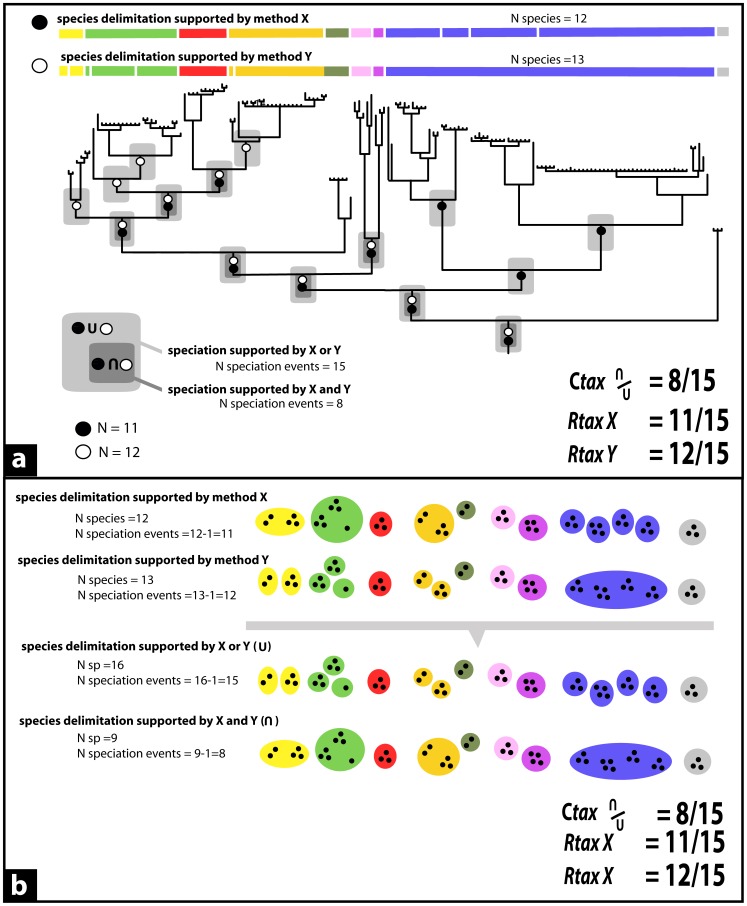
Calculation of the Relative taxonomic resolving power index (R*_tax_*) and Taxonomic index of congruence (C*_tax_*). Calculations are exemplified on two distinct species delimitation approaches (X and Y) supporting different taxonomies. For a better understanding, calculations are first exemplified on a tree-based taxonomy (a). Although these calculations are based on a underlying phylogenetic perspective, knowledge of tree topology is not mandatory to perform the calculations (b). In (a), speciation event hypotheses supported by the approaches X and Y are represented by black and white circles, respectively. The C*_tax_* between both approaches is defined as the ratio of the total number of speciation events congruently supported ( = *shared*) by both approaches (dark grey boxes), relative to the total number of speciation events cumulatively suggested by both approaches (in light grey boxes). The R*_tax_* of a given approach represents the proportion of speciation events that it supports ( = *single*), relative to the complete set of speciation events (set of boundaries cumulatively revealed by all the different approaches – only two approaches in this example). In (b), representing the same taxonomy, the same calculations have been performed without relying on a phylogenetic tree, the number of speciation events being indirectly inferred from the number of species (in a dichotomic species tree, N cladogenetic speciation events = N species – 1). Little black dots represent specimens or populations, and each colored oval represents a distinct species hypothesis according to the approach (or combination of approaches) used.

Species delimitation approaches have generally been compared on the basis of a single parameter, i.e., the total number of species identified (eg. [16], [35]; but see [43]). However, equal numbers of species do not necessarily imply equal species boundaries. For instance, Wiens and Penkrot [23] found that three species-delimitation approaches applied to a set of spiny lizard populations coincided to divide these in five species, but only two of these five species had the same limits across the three approaches. With increasing numbers of species in test data sets, and an increasing amount of available species delimitation methods, a case-by-case comparison becomes unfeasible. This hampers the assessment of quantitative (resolving power) and qualitative (reliability of inferred species boundaries) performance of species delimitation methods. We here propose two novel descriptive statistical tools to overcome this handicap (Figure 1):

i The *Relative Taxonomic Resolving Power Index* (R*_tax_*) quantifies the ability of a given approach to reveal a high number of potential candidate species, relatively to other approaches. Of the complete set of speciation event hypotheses cumulatively suggested by all approaches, the proportion supported by a given approach becomes its R*_tax_*:

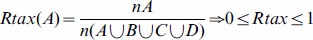
for an example with four approaches to be compared (*A, B, C, D*) with 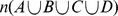 being the total number of possible speciation events revealed (i.e. supported by A and/or B and/or C and/or D). High values indicate high relative resolving power of a species delimitation approach, but do not necessarily imply reliability of the results: a high R*_tax_* indicates that an approach can alone retrieve all the species boundaries that have been independently identified by all the approaches, meaning a minimization of the beta-error (false negatives), but possibly a maximization of the alpha-error (false positives).

ii The *Taxonomic* index of congruence (C*_tax_*) allows the comparison of the congruence of taxonomies inferred by two different approaches. The C*_tax_* is the ratio of the number of speciation event hypotheses (pairwise species boundaries) congruently supported by the two approaches, relative to the total number of such hypotheses cumulatively supported by them

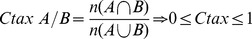
where A and B represent two different taxonomic approaches, *n*(A∩B) represent the total number of speciation event hypotheses congruently supported both by A and by B, and *n*(AUB), the total number of speciation event hypotheses congruently supported by A and/or by B. Thus, the highest value (C*_tax_* = 1) indicates that both approaches give identical taxonomies, supporting exactly the same species hypotheses (thus suggesting a reduced alpha-error). If the index is low, this indicates incongruence between the approaches (underestimation, overestimation, or mis-estimation by at least one of them).

## Results

### Phylogeny

**Figure 2:**
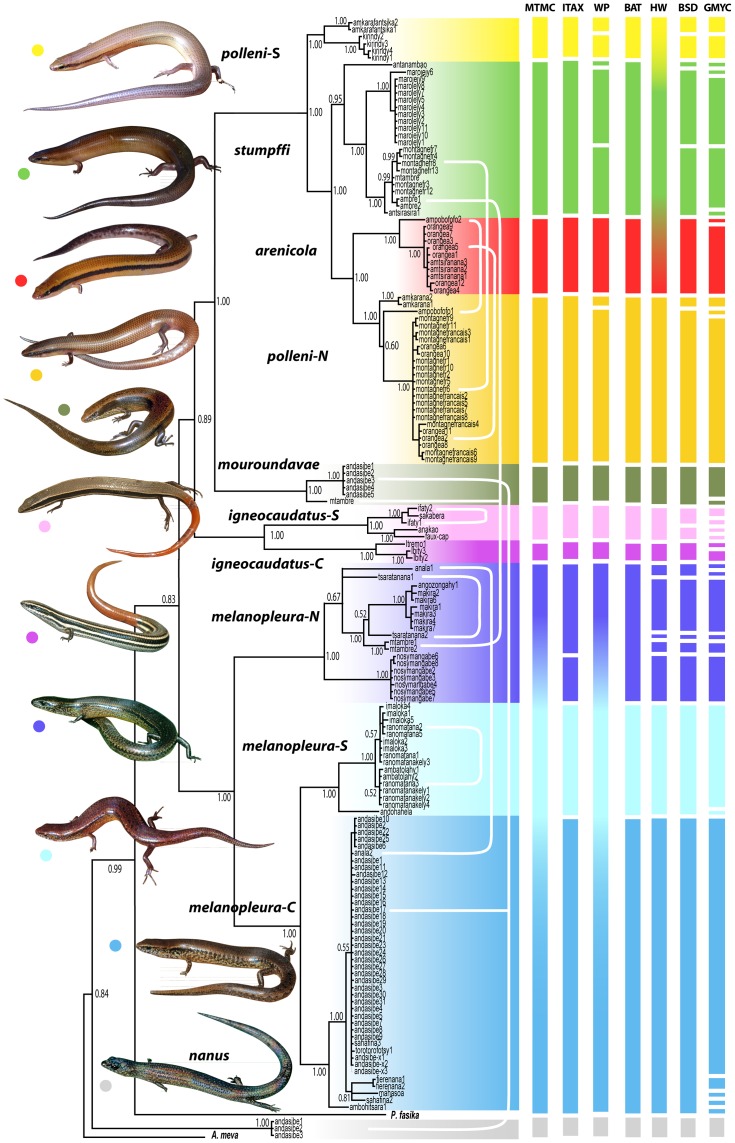
Summary of the taxonomies proposed for the genus *Madascincus* using different species delimitation approaches. Correspondences with clades are shown on the mtDNA gene tree (BI analysis of concatenated ND1and 16S sequences, posterior probabilities indicated for each node). The seven vertical multicolored bars represent alternative taxonomies, respectively supported by the Mitochondrial Tree – Morphological character Congruence (MTMC), Integrative Taxonomy (ITAX), Wiens-Penkrot (WP), Bayesian Assignment Test (BAT), Haploweb (HW), Bayesian Species Delimitation (BSD) and Generalized Mixed Yule-Coalescent (GMYC) approaches for species delimitation, each segment of these bars representing distinct species according to the respective approach. White lines connecting different samples in the phylogeny represent instances of sympatry between different clades.

The BI tree of the concatenated mitochondrial data set (16S and ND1) is overall relatively well supported (PP>0.95 for most of the nodes, Figure 2). It retrieves the polyphyly of *Madascincus polleni* as obtained previously based on one mitochondrial (ND1) and one nuclear (RAG2) marker [47] and supports the monophyly of the other six nominal species (PP = 1.0 for all of them). For convenience we selected eleven main clades (all of them supported by PP = 1.0) to be consistently represented by different colors across the present article, thereby facilitating discussion and visual comparison across species delimitations resulting from the different methods employed: *M. polleni* (1) from northern and (2) from southern Madagascar (*polleni-N* and *-S*), (3) *M. stumpffi*, (4) *M. arenicola*, (5) *M. mouroundavae*, *M. igneocaudatus* (6) from the southern coastal lowlands and (7) from the central mountains (*igneocaudatus-S* and *-C*), *M. melanopleura* (8) from northern, (9) from central and (10) from southern Madagascar (*melanopleura-N*, *-C* and *-S*) and (11) *M. nanus*.

The tree derived from the concatenated nuclear data set is congruent in topology with the mtDNA tree (cf. nDNA tree and each nuclear gene haplotype network in Files S6 and S11), with only two exceptions: (1) the relative positions of the *igneocaudatus* and *mouroundavae* clades are inverted and (2) the monophyly of *Madascincus* is recovered with exclusion of *Paracontias*. Analyses of the nDNA, mtDNA and combined data sets with different partition schemes (by codon and by gene and codon), and using coalescent species tree approaches, again confirmed the topology of the mtDNA tree, with variation affecting only the relative positions of the *igneocaudatus* and *mouroundavae* clades, and of the *stumpffi* vs. *polleni-S* clades. In the highly partitioned nDNA analyses (by codon, and by gene and codon) specimens of *polleni-S* did not form a monophyletic group, but this placement received no support (PP<0.5) (details in File S6). Species trees inferred by *BEAST agreed with the mtDNA phylogeny except the nDNA-only species tree placing *polleni-S* sister to *stumpffi*. As a consequence from the general congruence of mtDNA and nDNA phylogenies, we refute mtDNA introgression as a major theme in the evolutionary history of *Madascincus*.

### Species Delimitation

***MTMC*** **(** **Figure 2** **).** Morphological descriptive statistics are summarized in Table 1. On the basis of the encountered differences and the mtDNA tree, 9 morphologically diagnosable lineages were identified and considered as distinct species. This includes the recognition of two species within *Madascincus polleni* (*polleni-N* and *polleni-S*; *M. polleni* as currently understood being a polyphyletic unit) and the two sister lineages within *M. igneocaudatus* (*igneocaudatus-S* and *-C*). Given our decision to be most conservative in all approaches, all *melanopleura* specimens were merged in a single species.

**Table 1:** Summary of the morphological characters differentiating each pair of main clades of *Madascincus*.

	*nanus*	*igneo.-S*	*igneo.-C*	*mouroun.*	*melano.-N*	*melano.-C*	*melano.-S*	*polleni-N*	*polleni-S*	*stumpffi*
***arenicola***	F, T, VR, PR, MR,PN, FS, SO	F, N, PN, EW	F, MR, N,PN, EW	F, VR, PR, MR, PN,FS, EW	T, VR, PR, N, PN,FS, EW	VR, PR, PN,FS, EW	T, VR, PR, PN, FS, EW	VR	PN	MR
***nanus***	–	F, T, VR, PR,MR, FS, EW	F, T, VR, PR, MR, FS, SO, EW	F, T, VR, PR,MR, SO	T, MR, SO, EW	T, MR, SO, EW	T, MR, EW	F, T, VR, PR,MR, FS	F, T, VR, PR, MR,FS, SO	F, T, VR, PR, MR, SO
***igneocaudatus-S***	–	–	R	VR, PR, MR, N,FS, EW, R	F, T, VR, PR, FS	VR, PR, FS	VR, PR, FS	N, EW	EW	MR, N, EW
***igneocaudatus-C***	–	–	–	VR, PR, MR, N,FS, EW	F, T, VR, PR, FS	VR, PR, FS	VR, PR, FS	N, EW	EW	MR, N, EW
***mouroundavae***	–	–	–	–	F, T, MR, N, EW	VR, MR, EW	T, MR, EW	MR, FS	VR, PR, MR, FS	VR, PR
***melanopleura-N***	–	–	–	–	–	none	none	T, VR, N,FS, EW	T, VR, PR, FS, EW	T, VR, PR, MR, N, EW
***melanopleura-C***	–	–	–	–	–	–	none	T, VR, PR,FS, EW	VR, PR, FS, EW	VR, PR, MR, EW
***melanopleura-S***	–	–	–	–	–	–	–	T, VR, PR,FS, EW	T, VR, PR, FS, EW	VR, PR, MR, EW
***polleni-N***	–	–	–	–	–	–	–	–	VR	MR
***polleni-S***	–	–	–	–	–	–	–	–	–	MR

Only unambiguous diagnostic characters (eg. fixed character states for qualitative characters or non-overlapping values for meristic characters) are reported; See complete data in File S3. F: number of lamellae under 4th finger; T: number of lamellae under 4th toe; VR: number of ventral scale rows; PR: number of paravertebral scale rows; LR: number of longitudinal scale rows at mid–body; N: number of enlarged nuchal scales; PN: presence or absence of postnasal scales; FS: shape of the frontal scale; SO: position of the subocular scale; EW: aspect of the lower eyelid window; R: reproduction mode.

***ITAX*** **(** **Figures 2** **,** **3A** **).** The combination of the four criteria that were applicable for the species delimitation recognized the existence of overall 12 species, including two distinct species within *M. igneocaudatus* (*igneocaudatus-N* and -*C*) and four within *M. melanopleura* (the *melanopleura-C* and *-S* clades, plus two within the *melanopleura-N* clade). Compared to MTMC, the higher resolution within *melanopleura* was based on one example of syntopic occurrence without admixture among *melanopleura-N* and *-C*, and on absence of allele sharing in the majority of nuclear genes.

**Figure 3:**
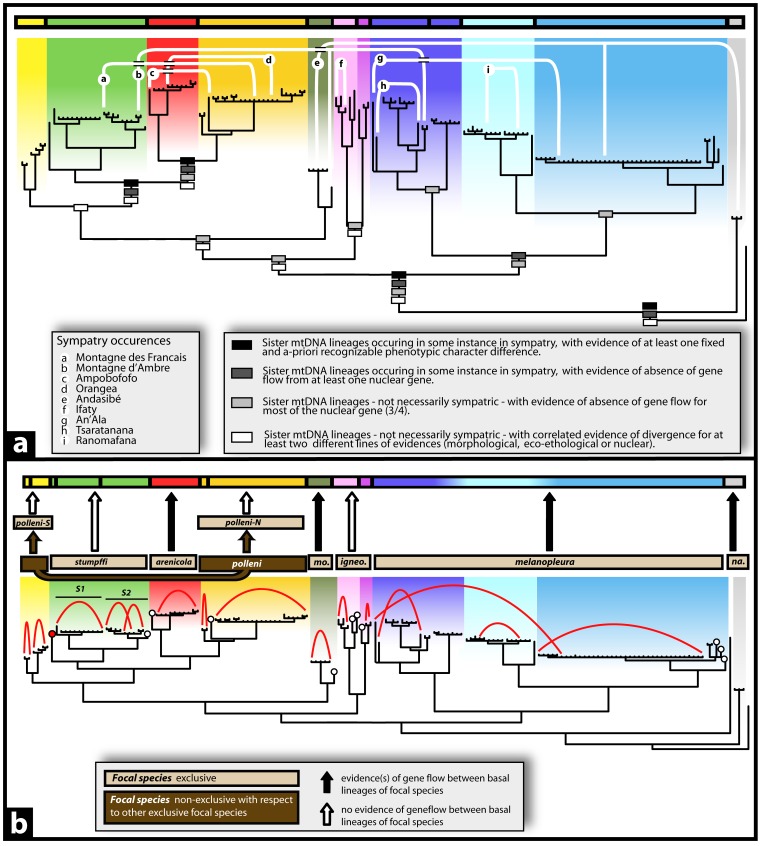
Results from the Integrative Taxonomic (ITAX) and Wiens and Penkrot (WP) approaches to species delimitation. Taxonomies are summarized above each figure by a horizontal multicolored bar, each segment representing a different species. A: Application of the ITAX protocol on the mtDNA gene tree. Four distinct criteria have been applied for speciation delimitation within the genus *Madascincus*. White lines connecting terminal taxa represent occurrences of sympatry (localities a-i) between major clades. B: Application of the WP protocol on the mtDNA gene tree. All the seven focal species tested represent exclusive haplotype lineages, with the exception of *M. polleni*. Two haplotypes have being considered as the minimal acceptable sampling to support the distinctiveness of a given species. Therefore, species revealed by the WP protocol that were represented by a single haplotype (white circle) were merged with their sister species. The unique sample of *M. stumpffi* from Antanambao (red circle) constitutes the only exception to this rule: this sample represents the sister lineage of two clades (Files S1 and S2) which are both well sampled and recovered as distinct species. Therefore, in accordance with the concept of phylogenetic species on which this protocol is based, the distinctiveness of the Antanambao sample as a third species has been validated. Red lines represent a selection of the most relevant instances of “gene flow” within each inferred species.

***WP*** **(** **Figures 2** **,** **3B** **).** According to the WP protocol 13 species were recognized. Six of the seven *a priori* focal species represented exclusive haplotype lineages. As the only exception *Madascincus polleni* was paraphyletic with respect to *M. arenicola* and *M. stumpffi.* For four focal species, some evidence of gene flow was evident between the basalmost lineages, supporting *M. arenicola*, *M. mouroundavae*, *M. melanopleura* and *M. nanus* each as a single species. On the contrary no evidence of gene flow was detected between basal lineages of the three other focal species, suggesting the presence of three, two and four distinct species within *M. stumpffi*, *M. igneocaudatus* and *M. polleni* (two in the *polleni-S* and two in the *polleni-N* lineages), respectively.

**Figure 4:**
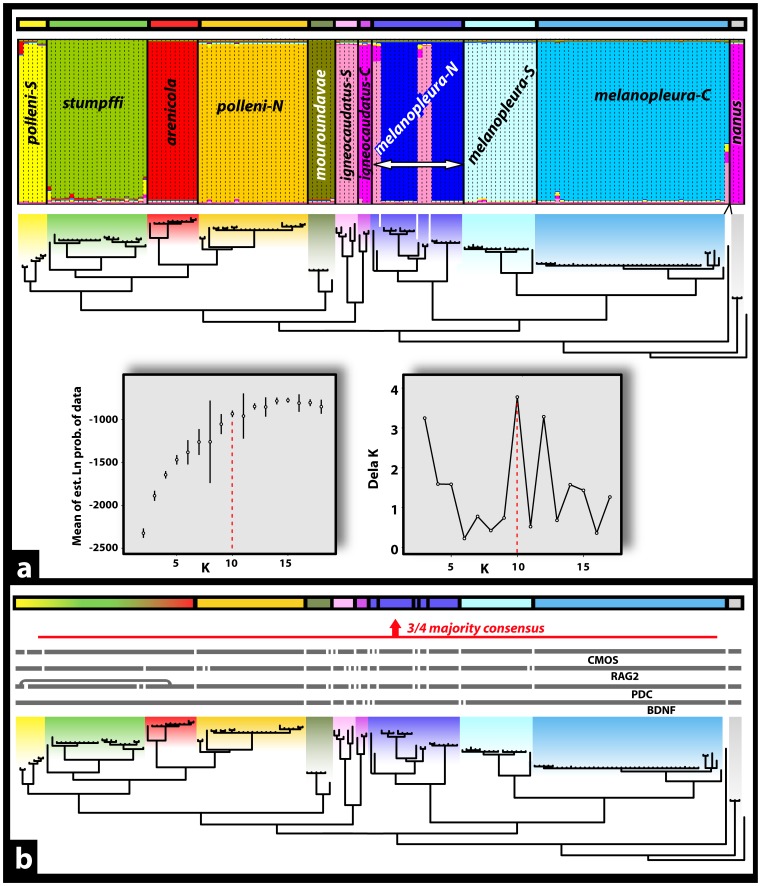
Results from the Bayesian Assignment Test (BAT) and the Haploweb (HW) approaches for species delimitation. Correspondence with clades is shown on the mtDNA gene tree (BI analysis of concatenated ND1 and 16S sequences). Taxonomies resulting from both approaches are summarized above each figure by a horizontal multicolored bar, each segment representing a species. A: Clusters in the nuclear STRUCTURE plot resulting from BAT, and their correspondence with clades in the mtDNA gene tree. Each cluster is marked with a different color with horizontal bars representing specimens and the proportion of a bar assigned to a single color representing the posterior probability that a specimen is assigned to that cluster. This can also be interpreted as the percentage of a specimen’s genome derived from that particular genetic cluster. mtDNA clades not mapped to the assignment plot represent out-group samples. Graphics in grey boxes represent calculations for various K values in STRUCTURE analysis of the nuclear data (ten replicates): left, the mean of the estimated log probability of the data for K = 3 to 18 ; right, ΔK values for K = 2 to 17. B: For each marker, single locus fields for recombination (pools of co-occurring haplotypes) inferred from the HW approach are represented by distinct segments of the grey bars, each bar representing one of the four nuclear haploweb reconstruction (cf. File S11). Species delimitation is based on a majority consensus of these four haplowebs: two populations being only considered as distinct species if at least three markers out of a total of four congruently recognize them as distinct fields for recombination. Taxonomies resulting from both BAT and HW approaches are summarized above each figure by a horizontal multicolor bar, each segment representing a species. Note that only haplotype sharing and not the connections between haplotypes are taken into account for species delimitation.

***BAT*** **(** **Figure 4A** **).** Plots of the estimated log probability of the data [log Pr(X|K)] for replicated STRUCTURE analyses revealed a general pattern of a plateau or decrease in values above K = 10 (Figure 4A, left grey box). Consistently, the calculations of ΔK produced a peak at K = 10 (Figure 4A, right grey box). A plot of individual membership coefficients for K = 10 revealed a high number of population clusters with average individual membership coefficients (i.e. posterior probabilities) greater than 0.95. Therefore, K = 10 appeared as a reasonable estimate of the upper level of population clustering within the genus *Madascincus*.

There is strong concordance between the nuclear clusters identified and the terminal mtDNA clades. Seven out of the 11 main mtDNA clades as previously defined mapped exclusively on one of the 10 nDNA clusters. Specimens of the *igneocaudatus-C* mtDNA lineage had nDNA STRUCTURE assignments identical to specimens of *M. nanus.* Due to their highly divergent morphology and important phylogenetic differentiation there is however no doubt that these are different species. We therefore interpret this result as a methodological artifact and assign *nanus* and *igneocaudatus-C* each to a distinct species. It is less straightforward to interpret the nDNA heterogeneity observed within the *melanopleura-N* lineage: while most specimens are assigned to a cluster exclusive to *melanopleura*-N, some are assigned to *igneocaudatus-S.* Also in this case, the high morphological differences between *M. igneocaudatus* and *M. melanopleura* suggest a methodological artifact. Moreover, complementary analyses realised on the *melanopleura* clade subset support also the homogeneity of the *melanopleura*-N clade, the optimal clustering obtained revealing the existence of only 3 well-discriminated populations fitting with the three main mtDNA clades, i.e. *melanopleura*-N, -C and –S, see File S7). Following a conservative approach we manually overruled this probable artifact and assigned all of the *melanopleura*-N specimens to a single species as they form a morphologically homogeneous unit, a single homogeneous cluster in the subset separated analysis and a monophyletic lineage strongly supported both by the mitochondrial and by the nuclear phylogenetic tree. Overall, the “corrected” BAT approach thus recognized the existence of 11 species.

#### *HW* (Figure 4B)

In total, 11 different single-locus fields for recombinations were identified for BDNF, 18 for CMOS, 18 for PDC and 21 for RAG2; details in File S11. The majority consensus identified 13 distinct species (multiple-locus fields for recombination *sensu* Flot et al. [32] supported by at least three of the four markers).

#### *BSD* (Figure 5A)

The BSD analysis suggested 22, 24 and 20 species under the first, second and third combination of priors (both θ and τ_o = _0.1 / both θ and τ_o_ = 0.001 / θ = 0.1 and τ_o = _0.001). The three approaches congruently supported 19 species, including the splitting of *M. polleni*, *M. stumpffi, M. igneocaudatus* and *M. melanopleura* into four, three, three and seven distinct species, respectively.

#### *GMYC* (Figure 5B)

**Figure 5:**
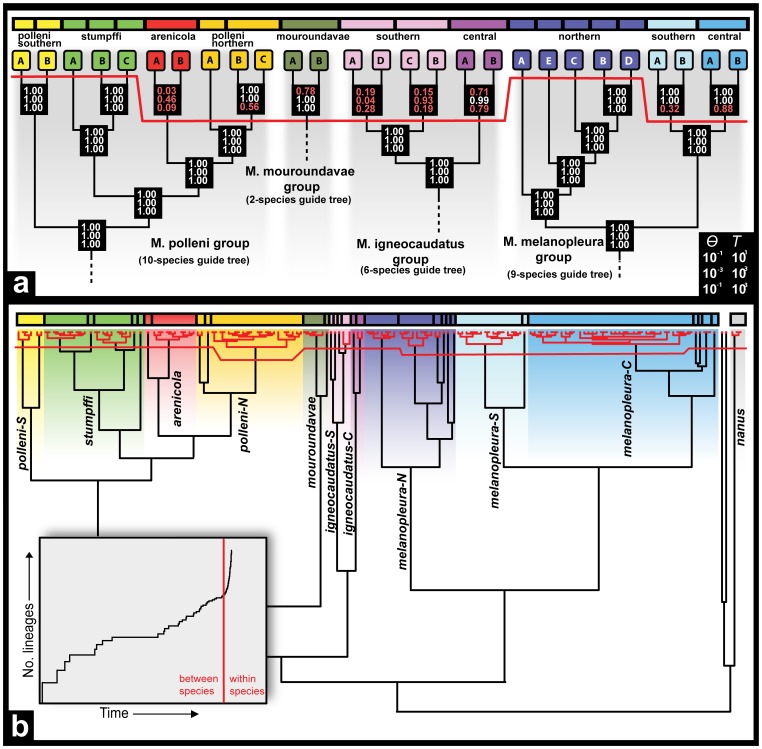
Results from the Bayesian Species Delimitation (BSD) and Generalized Mixed Yule-Coalescent (GMYC) approaches. Taxonomies resulting from both species delimitation approaches are summarized above each figure by a horizontal multicolored bar, each segment representing a different species. A: Guide trees used for each of the four BSD analyses (separately for the *M. polleni*, *M. mouroundavae*, *M. igneocaudatus* and *M. melanopleura* groups and assuming ten, two, six and nine species respectively (colored squares), cf. File S10 for details) are presented, with speciation probabilities provided for each node under each combination of priors for θ and τ_o_ (top, prior means = 0.1; middle, prior means = 0.001; bottom, prior mean θ = 0.1, prior mean τ_o = _0.001). Only speciation events simultaneously supported by probabilities superior or equal to 0.99 for all three combinations of priors were considered to be relevant for species delimitation. The distinction between supported and non supported speciation events is represented by a horizontal red line above which each lineage represent a single and distinct species. B: The distinction between “inter-specific” versus “intra-specific” nodes estimated by the GMYC approach is represented by a horizontal red line, and all intra-specific relationships are colored in red on the mtDNA chronogram. The graphic in the grey box represent the lineages-through-time plot based on the ultrametric tree obtained from all mitochondrial haplotypes. The sharp increase in branching rate, corresponding to the transition from interspecies to intraspecies branching events, is indicated by a red line.

The lineage-through-time plot (grey box in Figure 5B) indicated an approximately steady increase in lineage accumulation with a sharp increase in diversification rate toward the present. The single threshold model distinguished 34 putative species-level clades within the genus *Madascinscus.* The likelihood-ratio test gave a highly significant result (likelihood-ratio statistic was 48.22149, *P* = 1.9 e-10). The multiple threshold model distinguished 40 clades, also with a significant result of 51.01106 (*P* = 8.603742e-10). The multiple threshold method did not represent a significant improvement of the single threshold method (X^2^_6_ = 2.789), and we therefore retained the more conservative results from the single threshold model. This approach supported the splitting of almost all the nominal species within the genus, namely *M. polleni* (split into 5 distinct species), *M. stumpffi* (5), *M. arenicola* (2), *M. mouroundavae* (2) *M. igneocaudatus* (6), and *M. melanopleura* (13).

### Statistical Assessment and Comparison of Different Delimitation Approaches

**Figure 6:**
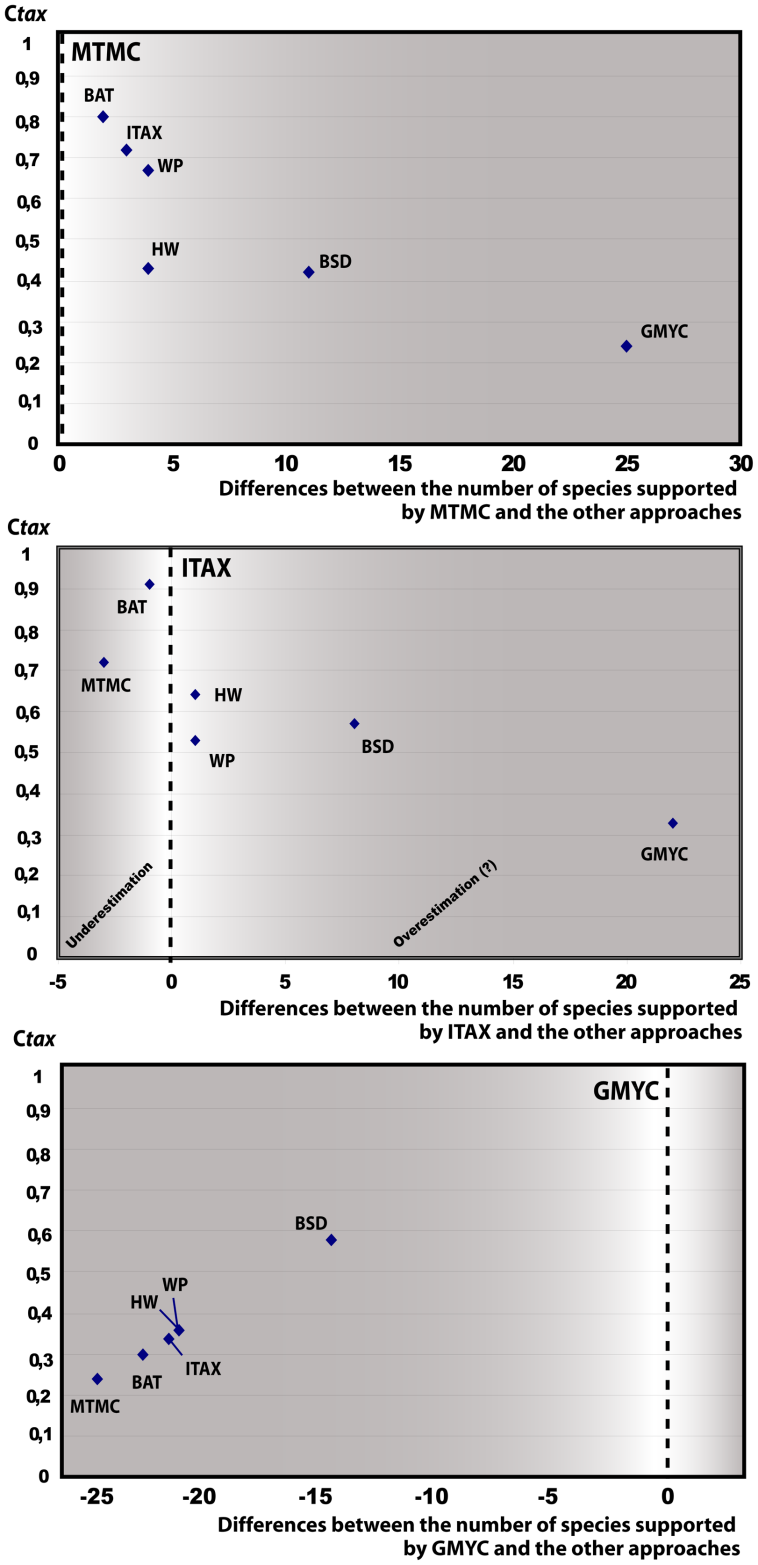
Comparison of the relative performance of species delimitation approaches relative to MTMC, ITAX and GMYC.

The seven approaches used to infer species limits within the genus *Madascincus* give strongly contrasting results (Figures 2 and 6, Table 2). Most inflationist is GMYC suggesting the existence of 34 distinct species, followed by BSD (n = 20), HW and WP (n = 13 for both approaches), ITAX (n = 12), BAT (n = 11) and the most conservative MTMC (n = 9). The resolving power of GMYC is maximal (R*_tax_* = 1.00), i.e., this method retrieves all species limits revealed by the other approaches together (plus additional ones). MTMC has a relatively low power of resolution (0.24), detecting only 24% of all species limits indicated by any method. Other approaches offer intermediate R*_tax_* values: 0.57 for BSD, 0.36 for both HW and WP, 0.33 for ITAX, and 0.30 for BAT.

**Table 2:** Summary of descriptive statistics employed to assess absolute and relative performance of the approach used for species delimitation.

	CTax							Mean Ctax	Rtax	Nb species	
	MTMC	BAT	HW	BSD	GYMC	ITAX	WP				
**MTMC**	–							0.52	0.24	9	
**BAT**	0.80	–						0.61	0.30	11	
**HW**	0.43	0.57	–					0.49	0.36	13	
**BSD**	0.42	0.53	0.63	–				0.56	0.57	20	
**GMYC**	0.24	0.30	0.36	0.57	–			0.35	1.00	34	
**ITAX**	0.72	0.91	0.64	0.57	0.33	–		0.62	0.33	12	
**WP**	0.67	0.57	0.33	0.63	0.36	0.53	–	0.51	0.36	13	

**Figure 7:**
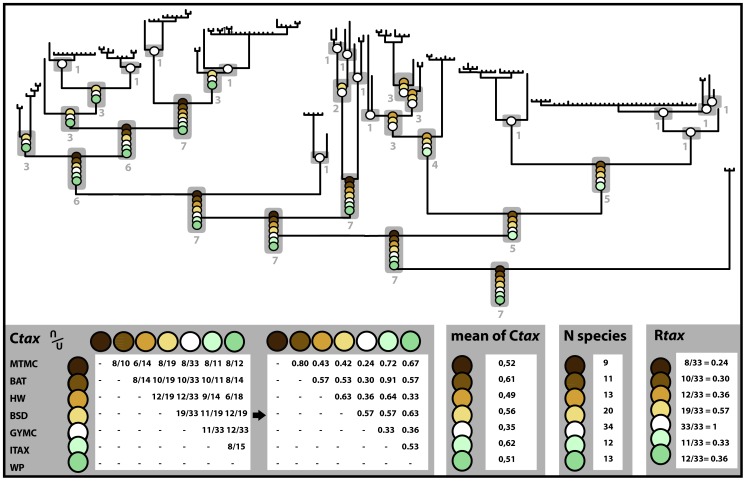
Relative taxonomic resolving power index (R*tax*) and Taxonomic index of congruence (C*tax*) calculation for each species delimitation approaches tested. Each putative speciation event inferred by all seven approaches (cf. Fig. 2) is reported for better visualization on the mtDNA tree topology (although these indices can also be understood without relying on a specific tree topology). Each color represents a distinct method. The R*tax* of a given approach represents the proportion of speciation events that are really supported by this approach, among the complete set of speciation event hypotheses (set of boundaries cumulatively revealed by different approaches. The C*tax* between two approaches is defined as the ratio of the total number of speciation events congruently supported by both approaches, to the total number of pairwise species boundaries cumulatively supported by both approaches. The mean C*tax* value (mean of all C*tax* values involving each of these approaches) and the number of species supported by each approaches is also presented.

C*tax*: Taxonomic index of congruence calculated for each pair of approaches. Mean C*tax*: Mean of all the C*tax* values obtained involving a given approach. R*tax*: Relative taxonomic resolving power index calculated for each approach; Nb species: total number of species supported by each approach (*cf.* Figure 7 for details).

The most congruent pair of approaches consists of BAT and ITAX with C*_tax_* = 0.91, i.e., 91% of all species limits inferred by the two methods are in agreement. The most incongruent pair is MTMC and GMYC with C*_tax_*_ = _0.24, and C*_tax_* values of the other pairwise comparisons range from 0.30–0.80.

Globally, ITAX is the most consensual approach in respect to all the others, as its mean C*_tax_* value (mean of all C*_tax_* values involving this approach) is 0.62, followed by BAT (0.61), BSD (0.56), MTMC (0.52), WP (0.51), HW (0.49) and GMYC which is the method with the lowest overall congruence (0.35).

## Discussion

### The Dilemma of Quality Assessment of Taxonomies

Not unexpectedly the various approaches to species delimitation applied here yielded highly different estimates of species numbers within *Madascincus,* between 9 and 34 species. Moreover, applying the new C*_tax_* metric proposed herein reveals that the various approaches also disagree in the placement of species boundaries (Figure 2, Table 2). When plotted on a phylogenetic tree (see Figure 7), most of these uncertainties are concentrated around the terminal nodes of the tree while at increasingly deeper nodes the congruence among approaches increases as well. These expected results are explained by the fact that lineages of older divergence had more times to accumulate differences in various sets of characters, whereas younger “speciation nodes” either did not yet lead to divergence in all character sets and to lineage sorting for all genes, or simply refer to conspecific lineages wrongly inferred to be species. All methods agree, however, on suggesting that *Madascincus* contains more species than currently recognized, and that at least two taxa represent new species (*polleni*-N and *igneocaudatus*-C clades). A tentative revision highlighting open taxonomic and nomenclatural issues is found in File S1.

Is it at all possible to define any species limits as correct or wrong relative to an alternative hypothesis? Species delimitation under the General Lineage or Evolutionary Species Concepts [8], [9], means identifying independent evolutionary lineages by applying certain Species Criteria (SC), but the results will differ depending on the SC applied. For example, strict application of the Biological SC will reject species status of such lineages that regularly hybridize along a narrow hybrid zone, although these are recognized by most other SC. Hence there is no objective way of comparatively assessing the quality of taxonomies that stringently apply different SC. An alternative means of quality control remains however available, that is, assessing whether a taxonomy indeed is based on a strict application of the SC that it claims to apply. In fact most of the species delimitation methods (GMYC, BSD, and others) implicitly claim to infer fulfillment or not of certain SC by applying algorithms. We therefore hold that it is possible to evaluate the performance of these methods relative to the underlying SC, i.e., to identify cases where species boundaries proposed are obviously erroneous by violating the underlying SC.

Related to the problem of evaluating taxonomies is a major unexplored and unsolved epistemological problem of alpha taxonomy under the evolutionary species concept, that of hypothesis falsification. A species hypothesis can be falsified in a Popperian framework only if formulated as one-species null hypothesis: it assumes a group of specimens being conspecific, and is rejected by evidence for the presence of more than one independent evolutionary lineages. Studies that explicitly refer to falsification of species hypotheses typically deal with such cases (e.g. [84]). The inverse case is more complex. A two-species null hypothesis according to which two clusters of specimens are two independent evolutionary lineages with their own evolutionary fate and historical tendencies [85], [86] can be tested only in the context of a particular SC, by expanding or reanalysing the initial data set and thereby possibly refuting the available evidence [87]. However under integrative taxonomy [12], [13], if one SC fails in supporting the two clusters of individuals as distinct species, this does not imply a falsification of the two-species hypothesis. This is because the critical evidence for species status of the two clusters might be found in another SC or, in practice, in another molecular marker, another morphological trait, or another line of evidence. There clearly is a void of studies analyzing this epistemological issue [87]. As a direct implication for our study, it is a complex endeavor to falsify the status of a certain lineage as separate species if proposed by a certain species delimitation approach, while it is more straightforward to identify cases in which an independent lineage has not been detected. Quantifying the alpha error of a taxonomy (excessive splitting) is thus more difficult than quantifying the beta error (excessive lumping). This leads us to strongly favor a conservative position in taxonomy given that errors originating by an exaggerated lumping are more likely to be detected and corrected by subsequent studies than those produces by inflationist approaches.

### Evaluating Species Delimitation Approaches in *Madascincus*

Taking the conceptual restrictions into account we first compare the results of all species delimitation approaches with MTMC as the closest approximation to current taxonomic practice. Second we evaluate all approaches relative to ITAX to assess the beta error (under-estimation) of the other methods, because ITAX is likely not over-estimating the real species diversity – it typically only accepts species hypotheses supported by strong evidence from at least one field of research [13]. Third, we discuss a number of highly implausible species hypotheses and use these as indicator for a substantial alpha error in some species delimitation approaches.

The MTMC approach was most conservative in proposing only 9 species in our *Madascincus* data set. We suggest that this mirrors the putative outcome of a classical taxonomical examination of the combined molecular-morphological data, searching for clear morphological differences among mitochondrial lineages. BAT, ITAX, WP, and HW yielded species numbers only slightly above the MTMC approach (Figure 6) but the C*_tax_* value of HW was very low, suggesting that this approach places species boundaries differently compared to current taxonomic practice. Two other approaches (BSD and GMYC) proposed many more species boundaries and also disagreed strongly with MTMC regarding the placement of these boundaries.

A comparison with ITAX yielded similar results, not unexpected given that C*_tax_* values are not independent among comparisons. Taking ITAX as a yardstick for a reliable taxonomic resolution, then among the software-based methods BAT performed best both in absolute species numbers and C*_tax_*, and WP, BSD and especially GMYC performed worst.

Close examination of the species boundaries proposed by the various methods and taking into account geographic and biological data (as in ITAX) reveals several unambiguous cases of underestimation of species diversity (beta-error or false negative). According to the HW approach, the clade (*polleni-N* + *polleni-S* + *stumpffi* + *arenicola*) represent a single species, but these four sub-clades show a clearly divergent morphology, distinct haplotypes for the fast-evolving markers (mtDNA and RAG2), and there are at least three instances of sympatry within this group (*arenicola* and *polleni-N* sympatric in Ampombofofo and in Orangea, and *stumpffi* and *polleni-N* in Montagne des Français, Figure 3A). As a second example, the MTMC approach is inherently unable to differentiate morphologically cryptic species if they are sister groups. MTMC therefore considers the three main clades of *melanopleura* (N, S and C) as a single species due to the absence of diagnostic morphological differences, whereas the existence of one case of sympatry observed between the clades *melanopleura-N* and *melanopleura-S* in An’Ala and the absence of nuclear haplotype sharing suggests they are independent evolutionary lineages.

Our results also include several examples that we interpret as obvious over-estimation of species diversity (alpha-error or false positives). GMYC suggests overall exaggerated species numbers, as can be exemplified in two cases: it identifies five species within the *melanopleura-C* clade, whereas the nuclear data set does not show any evidence of divergence between populations (all specimens of this clade belong to the same field for haplotype recombination and are assigned to the same BAT cluster; Figure 4B). GMYC also proposes four species within the *igneocaudatus-S* clade, whereas it is obvious that at least two of them (clades “ifaty2+sakabera” and “ifaty1”) are conspecific: both of these haplotype lineages are sympatric in Ifaty, present an extremely low mitochondrial divergence (p-dist. = 0.4–0.6% for 16S and 1.3% for ND1) and a similar morphology, and belong to the same field for haplotype recombination for three markers (BDNF, PDC and RAG2) out of a total of four. In the *stumpffi* clade, two approaches (BSD and GMYC) consider this taxon as a group of three and five distinct species respectively, whereas the nuclear data set includes all specimens in the same field for recombination for all analyzed markers (with only one exception for a single specimen from Antsirasira presenting a single and unshared haplotype for the PDC gene). Specimens of the Marojejy population are split by GMYC into two distinct species although both groups are sympatric, identical in morphology and nuclear alleles, and have only a low mitochondrial divergence (p-dist. = 1.1 % for 16S and 0.8% for ND1).

In our comparison, GMYC, a single-locus approach, performed worst in terms of suggesting numerous species-level units that objectively were in error, but multi-locus approaches were not necessarily superior: also BSD and HW were highly incongruent with ITAX and MTMC, and thus with current taxonomic practice.

### Causes and Consequences of Incongruent Species Delimitation

According to our *Madascincus* case study, BAT stands out among the species delimitation methods as being most congruent with current integrative taxonomic practice although the assignment tests themselves produced some obviously erroneous clusters that reflect problems of the STRUCTURE program. This software is known to sometimes provide biologically meaningless overestimates of the underlying populations, and similar problems might be inherent to other population genetic clustering software as well [88]–[90].

Besides these methodological issues, performance of BAT and other methods is certainly influenced by the variability of the markers used. Of the nuclear genes applied herein, BDNF is a highly conserved gene and therefore linked many clearly differentiated species into a single field for recombination. Choosing only such conserved nuclear genes, or conversely, markers with very high substitution rates such as microsatellite sequences or highly variable SNP positions, would heavily affect the outcome of the HW, BAT and BSD approaches. For instance, SNPs allow for identifying very young sympatric species of cichlid fishes [91] but also distinguish allopatric populations that would typically not be considered as species. Similarly, microsatellite markers distinguish clusters of speciating specimens or populations (e.g., [92]) that probably do not represent evolutionary units of own historical fate, and are not considered as distinct species by taxonomists. Coalescence simulations and tests with a wide variety of markers in taxa accessible to genome-wide assessments of genetic diversity could help to select an ideal set of markers for taxonomic purposes.

Several of the approaches as applied herein might also be strongly influenced by sample size. If a finite number of specimens of a lineage are known, it is impossible to reliably infer fixed differences in a single character (e.g., [1]), and this problem obviously gets most accentuated with very low sample sizes. This applies to all methods that rely on identifying morphological differences among lineages, but also a purely molecular method such as HW is highly sensitive to sample size: the more specimens sequenced, the more likely that a rare allele will be detected that then might integrate a lineage into one field for recombination. For highly variable markers such as microsatellites, simulations have shown that high sample sizes of 20–30 specimens per population perform best to estimate genetic variability [90], [93], and large sample sizes are also a requirement for various approaches to species delimitation that require population-level sampling and therefore were not included in our comparison (e.g., [70], [71]; details in File S11).

However, rare species (known from a single or very few specimens) may be common in zoosystematics [94]. In our case study, this problem is exemplified by the *melanopleura-N* clade characterized by low sample sizes for most populations, and with strong disagreement among species delimitation approaches. Ignoring such undersampled lineages could lead to gross underestimates of species diversity whereas accepting them uncritically as distinct species could lead to overestimates. Given the trade-off between the number of specimens sampled per species and the number of characters [1], overcoming the problem of low specimen sampling by increased character sampling will be pivotal for a reliable algorithm-based species delimitation.

The discrepancies among species delimitation approaches revealed by our study, if extrapolated to other groups of organisms, will have important consequences to understand and conserve Madagascar’s biota. Species ranges are the basis to assess conservation priorities in this biodiversity hotspot [48], and it therefore is, for instance, relevant whether *melanopleura-C* is considered as a single, widespread species occurring in numerous protected areas, or split into five species as suggested by GMYC. Obviously, GMYC is a valuable method to objectively define major mitochondrial phylogroups but at least in lizards does not serve an accurate species delimitation. The five species of partly very small ranges defined by this approach within *melanopleura-C* probably not constitute evolutionary lineages given that they are defined by mtDNA haplotypes partly occurring in the same population, yet their recognition would divert unwarranted conservation efforts towards certain regions of the island [95].

Madagascar’s unique biota has also attracted a wealth of studies on biogeography and species formation. Claims have been made that many species in Madagascar are characterized by particularly small ranges [96] but it remains unstudied whether this is really different from other regions in the tropics [97]. Again, testing such macroecological questions relies on species being comparable and biologically meaningful entities and thus on the accurateness of species delimitation. Given that the GMYC approach has been widely used in screening the mitochondrial diversity of Madagascar’s insects (e.g., [35]), we emphasize that care must be taken when translating this and any software-based species delimitation into actual taxonomies and basing evolutionary, biogeographic and conservation assessments on these.

### Perspectives for Applying Automated Species Delimitation

In the course of the last three decades, systematic biology has experienced major conceptual and methodological advances. These have especially revolutionized phylogenetic inference, the fast accumulation of DNA sequences triggering the development of novel approaches and ever more sophisticated statistical algorithms to infer gene trees, species trees, timetrees, and patterns and rates of character evolution [98]–[103]. In comparison, alpha-taxonomy has long been neglected by evolutionary biologists and bioinformaticians [7].

If automated species delimitation is to become an integral part of a fast-track taxonomy protocol [5] rather than just an academic exercise, it will be crucial to develop user-friendly and streamlined software. Taxonomy-related software platforms so far are aimed at improving access to data on specimens, species, and distributions [104], but do not extend to species delimitation. Several of the approaches applied herein rely on a pipeline of largely unrelated programs. The BAT approach produced particularly accurate results but requires combining mtDNA phylogeny reconstruction with nDNA assignment tests and thus sequential use of totally unrelated software. Additional tools are needed to take also morphological data into account (e.g., [105]). No software so far allows integrating all kinds of taxonomic evidence: mitochondrial and nuclear DNA, categorical and continuous morphological characters, and geography. As stated above, sympatric occurrence of two lineages is of high importance for species delimitation: either (i) to assess conspecificity if the sympatric groups are supported by only a single marker or character but admixed in others, or (ii) to provide strong evidence for distinct species if a correlated differentiation in various independent markers is found.

The present study illustrates the trade-off between reliability and resolving power in taxonomy [13]. More sensitive methods are able to capture a maximum of potential species boundaries, but the complete set of species boundaries proposed is globally poorly reliable. On the contrary, more conservative methods seeking for congruence between independent lines of evidence reveal rather robust species boundaries but are quantitatively less informative. How this trade-off is influenced by differences between higher taxa, completeness of sampling and markers used remains remarkably understudied. A combination of further theoretical work, thorough case studies and simulations is needed to understand which approach is most efficiently and broadly applicable to species delimitation in a wide array of groups of organisms. Implementing such an approach, or more likely, a combination of approaches, into user-friendly software could be a milestone towards fast yet reliable species delimitation across taxonomic groups, thereby contributing to the much-needed acceleration of the inventory of life on our planet.

## Supporting Information

File S1 Tentative revision of the genus *Madascincus.*
(DOC)

File S2 List of specimens examined morphologically.
(DOC)

File S3 Original morphological measurements and meristic counts. a. Morphological characters and cephalic scales nomenclature. b. Comparison of the most relevant morphological characters examined for the present study.
(DOC)

File S4 Primer sequences and PCR conditions.
(DOC)

File S5 List of voucher specimens, GenBank accession numbers, and localities.
(DOC)

File S6 Phylogenetic results: complementary analyses. Bayesian Inference analyses of phylogeny carried out under different partition schemes to understand whether these would influence the general topology of the *Madascincus* phylogenetic tree and thus reveal flaws in the guide tree used for the BAT, WP and GMYC approaches. a. Comparison of phylogenetic trees based on nDNA and mtDNA data. b. Bayesian Inference tree of the mtDNA data set. c. Bayesian Inference tree of the nDNA data set under a partition scheme with 1st, 2nd and 3rd codon position (merged for all nuclear genes) defined as separate partitions. d. Bayesian Inference tree of the nDNA data set under a partition scheme with 1st, 2nd and 3rd codon position (separately for all nuclear genes) defined as separate partitions. e. Species trees (cladogram view) calculated with *BEAST, based on the combined mtDNA and nDNA data (above), and the combined nDNA data only (under). f. Species trees (cladogram view) calculated with *BEAST, based on the combined nDNA data, with individuals combined to terminal taxa on the basis of the analysis with STRUCTURE.
(DOC)

File S7 Bayesian Assignment tests: complementary analyses. a. STRUCTURE analyses based on complete dataset (all taxa) and data subsets, with subsequent solutions of higher K. b. Taxonomic congruence (Ctax) of the corrected and uncorrected BAT results.
(DOC)

File S8 Workflow and application of the ITAX protocol.
(DOC)

File S9 Workflow and application of the WP protocol.
(DOC)

File S10 Partitioning and combination of priors for Bayesian Species Delimitation (BSD) analysis.
(DOC)

File S11 Haploweb reconstructions for the four nuclear genes (BDNF, PDC, CMOS and RAG2).
(DOC)
