# Supplementary material for: New Metrics for Comparison of Taxonomies Reveal Striking Discrepancies among Species Delimitation Methods in Madascincus Lizards
Source: PLoS One. 2013 Jul 12;8(7):e68242. doi: 10.1371/journal.pone.0068242 (PMC3710018; doi:10.1371/journal.pone.0068242)
Supplement: File S1 — Tentative revision of the genus Madascincus. (DOC) [file pone.0068242.s001.doc]

**S1. Tentative revision of the genus *Madascincus***

Species names used in this manuscript largely followed current taxonomy but avoided the use of names that cannot be reliably assigned to a given clade due to missing information (e.g., absence of molecular data from the type localities). This implied provisionally merging *M. ankodabensis*, *M. minutus,* and *M. vulsini* with *M. melanopleura;* *M. intermedius* with *M. polleni* (*cf.* Miralles et al. 2011a); and a candidate species, referred as *Madascincus* sp. “baeus” by Glaw and Vences 2007 with *M. nanus*.

Despite relevant incongruences between all the species delimitation approaches assessed herein, all of them agree on the fact that the species diversity within the genus Madascincus is globally underestimated, and that at least two taxa represent undescribed species (polleni-N and igneocaudatus-C clades). We consider ITAX to represent the most consensual approach given that species boundaries inferred by this approach are exclusively based on highly reliable biological evidence for speciation. This method is therefore minimizing the risk of false positives, and thus favors taxonomic stability.

In total, the ITAX recognized 12 distinct species within our sampling. Madascincus arenicola, M. mouroundavae, M. nanus and M. stumpffi, must be treated as distinct species. According to type examinations and/or type localities, and in application of principles of nomenclature (ICZN 1999), M. polleni (Grandidier, 1869) – type locality (TL): Morondava – should be restricted to the polleni-S clade (including M. intermedius (Boettger, 1913) – TL: “Majunga” = Mahajanga – as a junior synonym), whereas the clade polleni-N represents a new undescribed species. Madascincus igneocaudatus (Grandidier, 1867) – TL: “Tuléar” (= Toliara) – should be restricted to the igneocaudatus-S clade, whereas the clade igneocaudatus-C represents also a new undescribed species. Madascincus melanopleura (Günther, 1877) – TL: “Anzahamaru” (close to Mahanoro in the central eastern lowlands – should be restricted to the melanopleura-C clade (including M. vulsini (Barbour, 1918) – TL: “eastern forest between Tamatave and Tananarive” – as a junior synonym), and M. ankodabensis (Angel, 1930) – TL: “Ankodabe”– should be applied to the melanopleura-S clade. In contrast, the taxonomy of the melanopleura-N clade remains complex. The ITAX approach suggests the existence of at least two genetically distinct but morphologically cryptic species within this group, whereas other approaches suggest up to six cryptic species, all occurring in allopatry but partly in close spatial proximity. A more complete sampling (both in term of number of localities and number of samples per populations) is essential to investigate more into detail the taxonomy of this complex taxon. Due to sampling gaps we are not able to determine to which of these lineages the name M. minutus (Raxworthy and Nussbaum, 1993) – TL: “Manongarivo” – should be assigned and we therefore propose conservatively to consider the whole melanopleura-N group as a single species M. minutus, pending a more detailed taxonomic revision of the genus.

**References:**

Glaw F, Vences M (2007) A Field Guide to the Amphibians and Reptiles of Madagascar. Third edition. Cologne, Vences and Glaw Verlag, 496 pp. S20.

ICZN (1999) International Code of Zoological Nomenclature, 4^th^ edn. London: International Trust for Zoological Nomenclature, The Natural History Museum.

**Miralles A**, Kölhler J, Glaw F, Vences M (2011a) A molecular phylogeny of the *Madascincus polleni* species complex, with description of a new species of scincid lizard from the coastal dune area of northern Madagascar. Zootaxa. 2876:1–16.
