# Supplementary material for: New Metrics for Comparison of Taxonomies Reveal Striking Discrepancies among Species Delimitation Methods in Madascincus Lizards
Source: PLoS One. 2013 Jul 12;8(7):e68242. doi: 10.1371/journal.pone.0068242 (PMC3710018; doi:10.1371/journal.pone.0068242)
Supplement: File S2 — List of specimens examined morphologically. (DOC) [file pone.0068242.s002.doc]

**S2. List of specimens examined morphologically**

***Madascincus arenicola*. ─** Antsiranana Province: ZSM 1565/2008 (FGZC 1703, holotype of *Madascincus arenicola* Miralles, Köhler, Glaw & Vences 2011), “Baie des Sakalava (ca. 5 km SE Ramena), 12°16'34'' S, 49°23'24'' E”, 25 m a.s.l. , collected on 20 February 2008 by S. Megson. ZSM 1564/2008 (FGZC 1922, paratype), Ampombofofo region (Frontier base camp), 12°05'53'' S, 49°19'49'' E, 28 m a.s.l., collected on 10 June 2006 by S. Megson; ZSM 2076/2007 (FGZC 1031, paratype), UADBA uncatalogued (FGZC 1029, 1030, paratypes), Baie des Dunes (E Ramena), 12°14'43'' S, 49°22'53'' E, 14 m a.s.l., collected on 23 February 2007 by P. Bora, H. Enting, F. Glaw, A. Knoll and J. Köhler; ZSM 1566–1569/2008 (FGZC 1743, 1744, 1767, 1797, paratypes), Baie des Sakalava (ca. 5 km SE Ramena), 12°16'34'' S, 49°23'24'' E, 25 m a.s.l., collected from 21 to 23 February 2008 by S. Megson. All localities within Antsiranana Province, northern Madagascar.

***Madascincus igneocaudatus* complex. ─ Southern clade:** Toliara province: MNHN1980-1203 to -1213, Saraondrey, Tsihombe region, 10 km NE Benonoka, coll. by Domergue and Razafy; MNHN1980-1214 Benonoka, 10 km NW Faux Cap, coll by Domergue and Razafy; MNHN1980-1215 Analamisaka, 30 km N Cap Ste Marie, coll. by Domergue; MNHN1980-1216 Aujiso, 20 km E Faux Cap, coll. by Domergue; MNHN1990-1598, -1599 Ihotry (South-West), coll. by Domergue; ZSM 1602/2010, (ZCMV 13012) Ifaty Mangily Reserve (23° 07’ 22.05’’S, 43°36’34.02’’E), coll. by Crottini; ZCMV 12819, Ifaty, dry forest (23°07’35’’S, 43°38’02’’E), coll. by Miralles and Ratsoavina; ZCMV 12826, Sakabera, mouth of the Fiherenana river (23°18'11’’S, 43°39'31’’E), coll. by Miralles and Ratsoavina; ZSM 1600/2010 (ZCMV 12888) Anakao, coastal dunes (23°39’19’’S, 43°39’00’’E), coll. by Miralles and Ratsoavina; ZSM 1601/2010 (ZCMV 12897) Faux-Cap, coastal dunes (25°34’07’’S, 45°31’52’’E), coll. by Miralles and Ratsoavina. ***Central clade*:** Fianarantsoa province: MNHN1980-1217, Mont Ibity, coll. by Thérézien and Capuron. ZSM 518–520/2001 (MV 2001-441, -444, -445), Mont Ibity, coll. by M. Vences, D. Vieites, L. Raharivololoniaina and D. Rakotomalala in march 2001. ZSM 521/2001 (MV2001-611), Itremo (camp, 1648m), coll. by M. Vences, D. Vieites, L. Raharivololoniaina and D. Rakotomalala in march 2001.

***Madascincus melanopleura* complex. ─ Northern clade:** Antsiranana province: ZSM 398-401/2005 (ZCMV 2163, 2164, 2166, 2167), Nosy Mangabe, 50-100 m a.s.l., 15°30'S, 49°46'E, coll. by F. Glaw, M. Vences, R.D. Randrianiaina in 2005. ZSM 402/2005 (ZCMV 2027), Marojejy, "Camp Simpona", 1326 m a.s.l., 14°26.199'S, 49°44.601'E, coll. by F. Glaw, M. Vences, R.D. Randrianiaina in 2005. ZSM 2221/2007 (FGZC 1370), Montagne d'Ambre, F. Glaw, P. Bora, H. Enting, J. Köhler, I. Knoll in 2007. Toamasina province: ZSM 207/2006 (ZCMV 2481), An'Ala (forest camp), 889 m a.s.l., 18.91926°S, 48.48796°E, coll. by D.R. Vieites, M. Vences, F. Rabemananjara, P. Bora, C. Weldon, J. Patton in 2006. **Central clade:** Toamasina province: BM 1946.8.21.34 (holotype of Gongylus melanopleura Günther,1877), “Anzahamaru”. ZSM 221/2003, 223/2003, 224/2003 (FG/MV 2002-3078, 2002-3131, 2002-3130), Fierenana, coll. By Vences and collaborators in 2003; ZSM 525/2001, Andasibé, coll, by Vences & Vieites in 2001; ZSM 2-4/2005 (ZCMV 2232-2234), ZSM 14-28/2005 (ZCMV 2258, 2260, 2262-2269, 2271, 2273, 2274, 2279, 2280), ZSM 38/2005, ZSM 42/2005, Andasibé (pitfall) 939 m a.s.l., coll by R. Dolch- (Mitsinjo) in 2005. ZSM 333-335/2004 (FGZC 630-632), likely Andasibé (?). MNHN 1980-1195, -1196, 1983-882-885, Station forestière Périnet (= Andasibé). **Southern clade:** Fianarantsoa province: MNHN 1930-332 (holotype of *Scelotes ankodabensis* Angel, 1930), “Ankodabé”, coll. by Decary. ZSM 229/2003 (FG/MV 2002-630), Ranomafana-National parc ("bernhardi site"), coll. by F. Rabemananjara & P. Bora in 2003. ZSM 236/2003 (FG/MV 2002-625), Ambohitsara (E Ranomafana), coll. By F. Glaw, M. Puente, L. Raharivololoniaina, M. Thomas & D.R. Vieites in 2003. ZSM 353/2006, ZSM 354/2006 (ZCMV 2879, 3061), Ranomafana, Ambatolahy river, 915 m a.s.l., 21°14.632'S, 47°25.573'E, M. Vences, P. Bora, E. & T. Rajeriarison, T. Razafindrabe, C. Weldon, O. Verneau, L. du Preezand local collectors in 2006. ZSM 355/2006 (ZCMV 2907), Ranomafana, Ranomafanakely, 1134 m a.s.l., 21°14.921'S, 47°22.307'E, coll. by M. Vences, E. Rajeriarison, Y. Chiari, E. Balian in 2006. ZSM 356/2006 (ZCMV 2961), Ranomafana, Imaloka, 1020 m a.s.l., 21°14.527'S, 47°27.909'E, coll. by local collectors in 2006. Toliara province: MNHN 1901-222, Fort Dauphin, coll. by Allaud. ZSM 115/2004, Andohahela, (Camp 2); ca. 600m a.s.l., coll. byF. Glaw, M. Puente, M. Thomas & R. Randrianiaina. ZSM 395/2005 (FGZC 2323), Manantantely, ca. 20-100 m a.s.l., 24°59'S, 46°55'E, coll. by P. Bora, F. Glaw, M. Vences in 2005.

***Madascincus mouroundavae.*** **─** Antsiranana province: MNHN 1893-218 (lectotype of *Scelotes Bellyi* Mocquard, 1894), “Montagne d'Ambre”, coll. by Allaud & Belly. MNHN 1893-219, -220 (paralectotypes of *Scelotes Bellyi* Mocquard, 1894), Montagne d'Ambre, coll. by Allaud & Belly. Toliara province (?): MNHN 1895-211 (holoype of *Gongylus mouroundava*, Grandidier, 1872), “Morondava” (possibly an erroneous locality), coll. by Grandidier. Toamasina province: ZSM 6/2005, 12/2005, 13/2005, 31/2005 (ZCMV 2239, 2253, 2254, 2289), Andasibé (pitfall), 939 m. a.s.l., 18°56.169'S, 48°24.734'E, coll. by R. Dolch (Mitsinjo) in 2005.

***Madascincus nanus* complex. ─** Toamasina province: ZSM 1/2005 (ZCMV 2219, specimen referred under the name *Madascincus* sp. “*baeus*” by Glaw & Vences 2007) Andasibe (pitfall), 939 m, 18°56.169'S, 48°24.734'E, coll. by R. Dolch (Mitsinjo) in 2005. Majunga Province: MRSN R1803 (holotype of *Amphiglossus nanus* Andreone & Greer, 2002), “Anjanaharibe-Sud Massif, Analabe Valley, Campsite W1, Befandriana Fivondronana, Mahajanga Faritany (Majunga Province)”, 14846'S, 49827'E, 1000 m a.s.l., coll. by F. Andreone, H. Randriamahazo and J. E. Randrianirina in 1996; MRSN R1806 (paratype of *Amphiglossus nanus* Andreone & Greer, 2002), from the type locality (14846'S, 49826'E, 1250 m a.s.l.); Diégo Suarez province: R1870 (paratype of *Amphiglossus nanus* Andreone & Greer, 2002), Masoala Forest, Campsite 3 (Andasin'I Governera), Antalaha Fivondronana, Antsiranana Faritany, 15818.55'S, 5080.22' E, colll. by F. Andreone and J. E. Randrianirina in 1998. Unknown exact locality: BM 1946.8.21.23 (87.12.22.10, type of *Scelotes macrolepis* Boulenger, 1888), “*Madagascar*” (no exact type locality), coll. by R. Baron.

***Madascincus polleni* complex. ─ Northern clade:** Antsiranana province: ZSM 242/2004 (FGZC 474), 245/2004 (FGZC 480), Montagne des Français, 334 m a.s.l., coll. on 23-24.2.2004 by Glaw, Puente and Randrianiana; ZSM 1571/2008 (FGZC 1766), 1572/2008 (FGZC 1844), Baie des Sakalava (ca. 5 km SE Ramena), 28 m a.s.l., coll. on 22./26.2.2008 by Megson; ZSM 1573–1577/2008 (FGZC 1680, 1678, 1836, 1838, 1687), Montagne des Français (pitfall lines 1 & 5), coll. on 19./25.02.2008 by D'Cruze and native collectors; ZSM 259/2004, Montagne des Français, coll. on 18.-28.2.2004 by Glaw, Puente, Randrianiaina and Razafimanantsoa; ZSM 1570/2008 (FGZC 1917), Ampombofofo region (trapsite 5), 28 m a.s.l., coll. on 23.02.2007 by Mason; ZSM 1562–1563/2008 (FGZC 1658, 1827), Ankarana, near Petit Tsingy, 90 m a.s.l., coll. on 16./24.02.2008 by Franzen, Glaw, Köhler and Nagy; MNHN 1897-31, Diego Suarez; MNHN 1980-1169, Bemanevika, Plateau Bealarana. **Southern clade:** Mahajanga province: SMF 16027 (lectotype of *Scelotes intermedius* Boettger, 1913), NW Madagascar, “*Majunga*”, coll. in 1897 by Voeltzkow. ZSM 522/2001 (MV 2001-313), Ankarafantsika (Ampijoroa), coll. on 24.02.2001 by Vences, Vieites, Garcia, Raherisoa and Rasoamamonjinirina; ZSM 523–524/2001, Ankarafantsika, coll. 2.2001 by Vences, Vieites, Garcia, Raherisoa and Rasoamamonjinirina. Toliara province: MNHN 1895-210 (holotype of *Gongylus polleni* Grandidier, 1869), “*Morondava*”, coll. by Grandidier; MNHN 1980-1201, coll. by Domergue; MNHN 1984-168, Beroboka MNHN 1984-169, Tuléar; MNHN 1905-132, plain of the Fiherena, coll. 1869 by Geay. ZSM 1595-1599/2010 (n=5), (ZCMV 12723, 12755, 12914, 12915, 12916) Kirindy reserve (20°04’40.0’’S, 44°41’30.1’’E), coll. by Miralles, Rakotoarison and Rakotondramanana.

***Madascincus stumpffi*. ─** Antsiranana province: ZSM 2162/2007 (FGZC 1228), Forêt d'Ambre, ca. 4 km WSW Sakaramy, 470 m a.s.l., coll. on 12.3.2007 by Bora, Glaw and Köhler; ZSM 1559/2008 (MgF 061), Montagne des Français, ca. 1.8 km SW Andavakoera ("trapsite 1"), 200 m a.s.l., coll. on 01.12.2006 by E. Randriamalala; ZSM 1558/2008 (FGZC 3124), Forêt d'Ambre, ca. 5 km SW Sakaramy (trapsite), 479 m a.s.l., coll. on 19.02.2008 by D'Cruze; ZSM 1560–1561/2008 (FGZC 1772, 1823), Montagne des Français (pitfalls), coll. on 22.-24.02.2008 by native collectors; ZSM 0041/2005 (FGZC 2785), 0032/2005 (ZCMV 2031), 0033/2005 (ZCMV 2032), 0040/2005 (FGZC 2784), 0405/2005 (ZCMV 2034), 0404/2005 (ZCMV 2033), 0043/2005 (FGZC 2787), Marojejy, "Camp Mantella" (14°26.260´S, 49°46.533`E), 481 m a.s.l., coll on 16.02.2005 by Glaw, Vences and Radrianiaina. SMF 16019 (holotype of *Gonglylus stumpffi* Boettger, 1882 and lectotype of *Scelotes astrolabi boettgeri* Angel, 1941), “*Nossibé*”, coll. in 1881 by Reuter and Stumpff; MNHN 1884-587, Nossi-Bé, coll. by Deyrolles. Mahajanga province: ZSM 206/2003 (FG/MV 2002-2268), 5 km from Antambao, close to Maevatanana, road to Manongarivo, coll. on 31.01.2003, by Glaw, Randrianiaina, and Vences. Toamasina province: MNHN 1980-1200, Mananara, coll. in 1963 by Peyrieras.
