# Supplementary material for: New Metrics for Comparison of Taxonomies Reveal Striking Discrepancies among Species Delimitation Methods in Madascincus Lizards
Source: PLoS One. 2013 Jul 12;8(7):e68242. doi: 10.1371/journal.pone.0068242 (PMC3710018; doi:10.1371/journal.pone.0068242)
Supplement: File S3 — Original morphological measurements and meristic counts. a. Morphological characters and cephalic scales nomenclature. b. Comparison of the most relevant morphological characters examined for the present study. (DOC) [file pone.0068242.s003.doc]

**S3. Original morphological measurements and meristic counts.**

**S3a. Meristic, mensural and categorical morphological characters examined here are those routinely used in the taxonomy of Scincidae, such as scale counts, presence or absence of homologous scale fusions, or color patterns (cf. Andreone & Greer 2002, Miralles et al. 2011a,b,c; cf. the figure below for complete scale nomenclature). The ventral scales are counted in a single row from the postmentals to the preanal scales (both included in the count), with mental scale excluded and the paravertebrals, in a single row from the first scale posterior to a line connecting the posterior edges of the thighs held normal to the long axis of the body anteriorly to and including the nuchals. Nuchal scales are defined as enlarged scales of the nape, occupying transversally the place of two or more rows of dorsal cycloid scale. The frontal scale is considered *hourglass-shaped* when constricted by first supraocular, *bell-shaped* otherwise. Measurements of specimens were recorded to the nearest 0.1 mm using a dial caliper. Ranges are given for each meristic and mensural character, followed by the mean ± the standard deviation, with sample size in parentheses. For some bilateral characters, the sample size has been noted as the number of sides rather than specimens (which in those cases is then indicated after the sample size).**

| **.**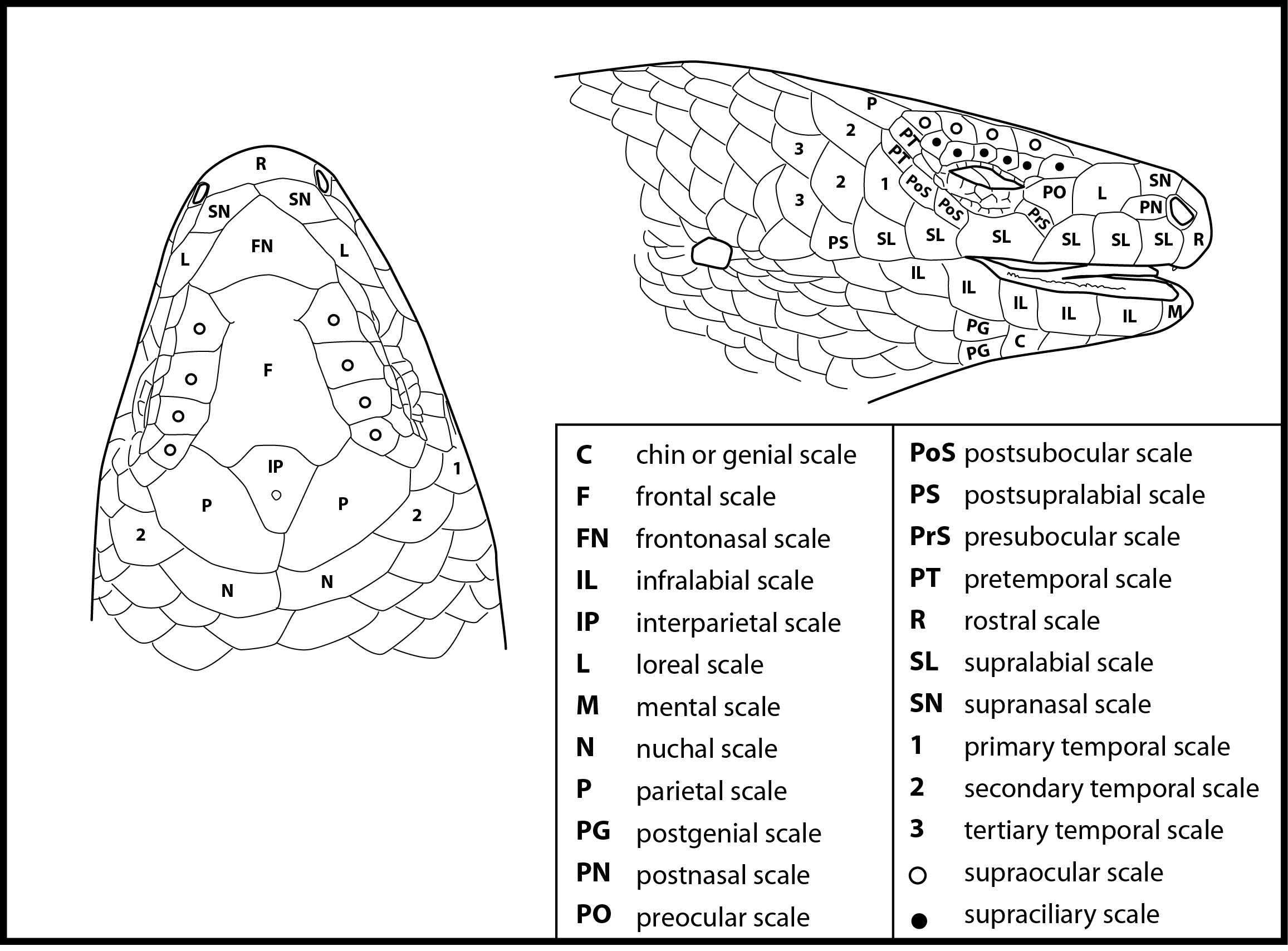 |
| --- |

**S3b.** Comparison of the most relevant morphological characters examined for the present study, plus additional data on the altitudinal distribution of main clades of *Madascincus*. Ranges are given for meristic and mensural character, followed by the mean ± the standard deviation, with sample size in parentheses. For some bilateral characters, the sample size has been noted as the number of sides rather than specimens. * data from litterature (Glaw & Vences 2007, Andreone & Greer 2002).

|  |  | ***arenicola*** | ***nanus*** | ***igneocaudatus*** | | *melanopleura* | | | ***mouroundavae*** | ***polleni*** | | ***stumpffi*** |
| --- | --- | --- | --- | --- | --- | --- | --- | --- | --- | --- | --- | --- |
|  |  |  |  | southern | central | northern | central | southern |  | northern | southern |  |
| N lamellae  under 4th finger | min–max:  mean ± SD:  n sides: | **6–7**  **6.4 ± 0.5**  **(11)** | **3–5**  **3.9 ± 0.6**  **(8)** | **8–11**  **9.0 ± 0.9**  **(50)** | **8–11**  **9.1 ± 0.9**  **(14)** | **5–7**  **6.0 ± 0.8**  **(13)** | **5–8**  **7.0 ± 0.6**  **(40)** | **5–8**  **6.3 ± 1.0**  **(9)** | **8–11**  **9.8 ± 0.7**  **(16)** | **7–8**  **7.6 ± 0.5**  **(22)** | **6–9**  **7.5 ± 0.7**  **(26)** | **6–9**  **7.3 ± 0.8**  **(27)** |
| N lamellae  under 4th toe | min–max:  mean ± SD:  n sides: | **16–19**  **17.5 ± 0.8**  **(13)** | **5–8**  **6.8 ± 1.3**  **(9)** | **15–22**  **18.1 ± 1.4**  **(54)** | **15–18**  **16.4 ± 1.3**  **(13)** | **9–13**  **11.3 ± 1.5**  **(10)** | **12–16**  **14.1 ± 1.2**  **(52)** | **12–15**  **13.8 ± 1.1**  **(12)** | **16–20**  **17.5 ± 1.2**  **(15)** | **18–23**  **20.6 ± 1.3**  **(20)** | **16–22**  **18.5 ± 1.5**  **(22)** | **15–20**  **17.9 ± 1.2**  **(28)** |
| N ventral  scale rows | min–max:  mean ± SD:  n: | **75–80**  **77.9 ± 1.6**  **(7)** | **52–60**  **57.6 ± 3.3**  **(5)** | **68–83**  **76.7 ± 4.4**  **(21)** | **73–78**  **75.7 ± 1.8**  **(7)** | **55–63**  **58.3 ± 3.0**  **(7)** | **56–61**  **58.8 ± 1.2**  **(27)** | **59–63**  **60.2 ± 1.5**  **(6)** | **63–66**  **64.3 ± 1.0**  **(8)** | **65–73**  **68.7 ± 2.1**  **(14)** | **74–78**  **75.8 ± 1.2**  **(12)** | **70–88**  **81. 3 ± 4.0**  **(16)** |
| N paravertebral  scale rows | min–max:  mean ± SD:  n: | **74–81**  **79.0 ± 2.3**  **(7)** | **50–57**  **53.6 ± 2.5**  **(5)** | **69–80**  **74.7 ± 3.0**  **(26)** | **71–79**  **74.6 ± 3.7**  **(7)** | **57–65**  **59.7 ± 3.4**  **(7)** | **51–62**  **55.9 ± 2.9**  **(28)** | **52–62**  **57.7 ± 3.1**  **(7)** | **60–65**  **62.6 ± 2.1**  **(8)** | **65–79**  **68.7 ± 3.3**  **(14)** | **71–81**  **77.9 ± 2.6**  **(13)** | **76–88**  **82.7 ± 3.2**  **(15)** |
| N longitudinal scale  rows at mid–body | min–max:  mean ± SD:  n: | **26**  **26.0 ± 0**  **(7)** | **18–20**  **19.6 ± 0.9**  **(5)** | **24–26**  **24.2 ± 0.6**  **(28)** | **22–24**  **23.3 ± 1.0**  **(7)** | **22–26**  **24.0 ± 1.2**  **(7)** | **24–26**  **24.1 ± 0.4**  **(27)** | **22–26**  **23.7 ± 1.5**  **(6)** | **28–30**  **29.0 ± 2.1**  **(8)** | **24–26**  **24.1 ± 0.5**  **(14)** | **24–26**  **25.4 ± 0.9**  **(13)** | **30-32**  **31.6 ± 0.8**  **(16)** |
| Enlarged nuchal  scales | absent :  one row :  two rows :  three rows :  four rows  n sides : | **42.9%**  **57.1%**  **–**  **–**  **–**  **(14)** | **–**  **40.0%**  **20.0%**  **40.0%**  **–**  **(10)** | **–**  **–**  **23.2%**  **71.4%**  **5.4%**  **(56)** | **–**  **–**  **21.4%**  **78.6%**  **–**  **(14)** | **–**  **–**  **28.6%**  **57.1%**  **14.3%**  **(14)** | **–**  **2%**  **50%**  **48%**  **–**  **(58)** | **–**  **7.1%**  **35.8%**  **57.1%**  **–**  **(14)** | **–**  **100%**  **–**  **–**  **–**  **(16)** | **92.3%**  **7.7%**  **–**  **–**  **–**  **(26)** | **56.3%**  **37.5%**  **6.2%**  **–**  **–**  **(22)** | **81.3%**  **18.7%**  **–**  **–**  **–**  **(32)** |
| Postnasal | Present  absent  n sides: | **–**  **100%**  **(14)** | **100%**  **–**  **(10)** | **100%**  **–**  **(56)** | **100%**  **–**  **(14)** | **100%**  **–**  **(14)** | **100%**  **–**  **(58)** | **100%**  **–**  **(14)** | **100%**  **–**  **(16)** | **89.3%**  **10.7%**  **(28)** | **100%**  **–**  **(26)** | **94.4%**  **5.6%**  **(36)** |
| Frontal and interparietal | Fused  Separated  n. | **–**  **100%**  **(7)** | **–**  **100%**  **(10)** | **–**  **100%**  **(28)** | **–**  **100%**  **(14)** | **–**  **100%**  **(7)** | **–**  **100%**  **(28)** | **–**  **100%**  **(7)** | **87.5%**  **12.5%**  **(8)** | **–**  **100%**  **(14)** | **–**  **100%**  **(13)** | **–**  **100%**  **(16)** |
| Frontal | bell shaped:  hour–glass shaped:  n: | **100%**  **–**  **(7)** | **–**  **100%**  **(10)** | **100%**  **–**  **(23)** | **100%**  **–**  **(12)** | **–**  **100%**  **(14)** | **–**  **100%**  **(29)** | **–**  **100%**  **(14)** | **–**  **100%**  **(8)** | **100%**  **–**  **(14)** | **100%**  **–**  **(13)** | **47.2%**  **52.8%**  **(18)** |
| Snout-vent length (mm) | max:  mean ± SD:  n: | **81.7**  **72.3 ± 6.1**  **(7)** | **33.6**  **27.8 ± 8.2**  **(6)** | **73.0**  **56.3 ± 11.6**  **(9)** | **54.2**  **52.3 ± 2.1**  **(4)** | **47.4**  **42.0 ± 5.1**  **(7)** | **53.5**  **49.5±2.5**  **(21)** | **50.5**  **48.0 ± 2.4**  **(5)** | **68.5**  **60.1 ± 9.6**  **(7)** | **61**  **54.9 ± 3.1**  **(14)** | **75**  **66.0 ± 7.1**  **(13)** | **114.0**  **89.6 ± 10.8**  **(14)** |
| Supraciliaries | five  Six  Seven  Eight  n sides | **–**  **92.9%**  **7.1%**  **–**  **(14)** | **–**  **66.6%**  **33.3%**  **–**  **(6)** | **–**  **98.2%**  **1.8%**  **–**  **(56)** | **7.1%**  **92.9%**  **–**  **–**  **(14)** | **7.1%**  **57.1%**  **35.8%**  **–**  **(14)** | **–**  **8.6%**  **79.3%**  **12.7%**  **(58)** | **–**  **35.7%**  **50.0%**  **14.3%**  **(14)** | **–**  **93.8%**  **6.2%**  **–**  **(16)** | **n/a** | **n/a** | **n/a** |
| Subocular | third SL:  Fourth SL:  n sides: | **–**  **100%**  **(14)** | **100%**  **–**  **(10)** | **1.8%**  **98.2%**  **(56)** | **–**  **100%**  **(14)** | **–**  **100%**  **(14)** | **–**  **100%**  **(58)** | **7.1%**  **92.9%**  **(14)** | **–**  **100%**  **(24)** | **3.6%**  **96.4%**  **(28)** | **–**  **100%**  **(24)** | **–**  **100%**  **(32)** |
| Lower eyelid window |  | **scaly** | **scaly** | **spec.** | **spec.** | **spec.** | **spec.** | **spec.** | **scaly** | **scaly** | **scaly** | **scaly** |
| Reproduction* |  | **?** | **?** | **vivi.** | **ovi.** | **?** | **?** | **?** | **ovi.** | **?** | **?** | **?** |
| Altidudinal range |  | **≤** 500m | 500 - 1500m | ≤ 500m | ≥ 1500 | ≤1000 | ≤1000 | ≤1000 | ≤1000 | ≥ 500m | ≥ 500m | ≥ 500m |

**References :**

Andreone F, Greer AE (2002) Malagasy scincid lizards: descriptions of nine new species, with notes on the morphology, reproduction and taxonomy of some previously described species (Reptilia, Squamata: Scincidae)*.* J. Zool. 258:139–181.

Glaw F, Vences M (2007) A Field Guide to the Amphibians and Reptiles of Madagascar. Third edition. Cologne, Vences and Glaw Verlag, 496 pp.

**Miralles A**, Kölhler J, Glaw F, Vences M (2011a) A molecular phylogeny of the *Madascincus polleni* species complex, with description of a new species of scincid lizard from the coastal dune area of northern Madagascar. Zootaxa. 2876:1–16.

**Miralles A**, Raselimanana AP, Rakotomalala D, Vences M, Vieites DR (2011b) A new large and colorful skink of the genus *Amphiglossus* from Madagascar revealed by morphology and multilocus molecular study. Zootaxa. 2918:47–67.

**Miralles A**, Köhler J, Vieites DR, Glaw F, Vences M (2011c) Developing hypotheses on rostral shield evolution in head-first digging squamates from a molecular phylogeny and new species of the genus *Paracontias* (Scincidae). Org. Diver. Evol. 11:135–150.
