# Supplementary material for: New Metrics for Comparison of Taxonomies Reveal Striking Discrepancies among Species Delimitation Methods in Madascincus Lizards
Source: PLoS One. 2013 Jul 12;8(7):e68242. doi: 10.1371/journal.pone.0068242 (PMC3710018; doi:10.1371/journal.pone.0068242)
Supplement: File S4 — Primer sequences and PCR conditions. (DOC) [file pone.0068242.s004.doc]

**S4. Primer sequences and PCR conditions**

PCR conditions start with temperature (in °C) of each step followed by the time in seconds. Data from: A: Palumbi et al. (1991), B: Schmitz et al. (2005), C: Leaché & Reeder (2002), D: Vieites et al. (2007), E: Han et al. (2004), F: Gamble et al. (2008), G: Bauer et al. (2007).

| Gene Primer | Sequence (5’ to 3’) | Source | PCR conditions |
| --- | --- | --- | --- |
| **16S rRNA** |  |  |  |
| 16S AL | CGC CTG TTT ATC AAA AAC AT | A | 94°(90s), [94°(45s), 55°(45s), 72°(90s) x 33], 72°(300s) |
| 16S BH | CCG GTC TGA ACT CAG ATC ACG T | A |  |
| **ND1** |  |  |  |
| intf2 | AAY CGV GCV CCW TTY GAC CTW ACA GA | B | 95°(120s), [95°(30s), 50°(30s), 72°(60s) x 40], 72°(600s) |
| tmet | TCG GGG TAT GGG CCC RAR AGC TT | C |  |
| **BDNF** |  |  |  |
| DRV_F1 | ACC ATC CTT TTC CTK ACT ATG G | D | 94°(120s), [94°(20s), 57°(45s), 72°(120s) x 39], 72°(600s) |
| DRV_R1 | CTA TCT TCC CCT TTT AAT GGT C | D |  |
| **CMOS** |  |  |  |
| C08 | GCT TGG TGT TCA ATA GAC TGG | E | 94°(180s), [94°(45s), 48°(45s), 72°(60s) x36], 72°(360s) |
| C09 | TTT GGG AGC ATC CAA AGT CTC 7 | E |  |
| **RAG2** |  |  |  |
| PY1-F | CCC TGA GTT TGG ATG CTG TAC TT | F | 94°(300s), [94°(30s), 55°(45s), 72°(60s) x 32], 72°(300s) |
| PY1-R | AAC TGC CTR TTG TCC CCT GGT AT | F |  |
| **PDC** |  |  |  |
| PHOF2 | AGA TGA GCA TGC AGG AGT ATG A | G | 95°(120s), [95°(35s), 50°(35s), 72°(150s) x 34], 72°(300s) |
| PHOR1 | TCC ACA TCC ACA GCA AAA AAC TCC T | G |  |

**References :**

Bauer AM, Silva A, Greenbaum E, Jackman TR (2007) A new species of day gecko from high elevation in Sri Lanka, with a preliminary phylogeny of Sri Lankan *Cnemaspis* (Reptilia: Squamata: Gekkonidae). Zoosyst. Evol. 83, 22–32.

Gamble T, Bauer AM, Greenbaum E, Jackman TR (2008). Evidence for Gondwanan vicariance in an ancient clade of gecko lizards. J. Biogeogr. 35, 88–104

# Han D, Zhou K, Bauer AM (2004) Phylogenetic relationships among gekkotan lizards inferred from C-*mos* nuclear DNA sequences and a new classification of the Gekkota. Biol. J. Linn. Soc. 83: 357–368.

Leaché AD, Reeder TW (2002) Molecular systematics of the eastern fence lizard (*Sceloporus undulatus*): A comparison of parsimony, likelihood, and Bayesian approaches. Syst. Biol. 51, 44–68.

Palumbi SR, Martin A, Romano S, MacMillan W, Stice L, Grabowski G (1991) The Simple Fool’s Guide to PCR (Ver. 2). University of Hawaii Press, Honolulu.

Schmitz A, Brandley MC, Mausfeld P, Vences M, Glaw F, Nussbaum RA, Reeder TW (2005) Opening the black box: phylogenetics and morphological evolution of the Malagasy fossorial lizards of the subfamily ‘‘Scincinae’’. Mol. Phyl. Evol. 34:118–133.

Vieites DR, Min MS, Wake DB (2007) Rapid diversification and dispersal during periods of global warming by plethodontid salamanders. Proc. Natl. Acad. Sci. USA 104, 19903–19907.
