# Supplementary material for: New Metrics for Comparison of Taxonomies Reveal Striking Discrepancies among Species Delimitation Methods in Madascincus Lizards
Source: PLoS One. 2013 Jul 12;8(7):e68242. doi: 10.1371/journal.pone.0068242 (PMC3710018; doi:10.1371/journal.pone.0068242)
Supplement: File S5 — List of voucher specimens, GenBank accession numbers, and localities. (DOC) [file pone.0068242.s005.doc]

**S5. List of voucher specimens, GenBank accession numbers, and localities**

GenBank accession numbers of previous studies are in bold (Miralles et al. 2011a,b,c) and missing data represented by N/A.

| Species | voucher  (sample) | Locality |  | | Sequences | |  | |
| --- | --- | --- | --- | --- | --- | --- | --- | --- |
|  | **N tot = 156** |  | BDNF | CMOS | PDC | ND1 | RAG2 | 16S |
| ***M. arenicola*** | (n=11) |  |  |  |  |  |  |  |
| ampobofofo2 | ZSM 1564/2008  (FGZC 1922) | Ampombofofo-region  (frontier base camp), 28m | JQ008056 | JQ008212 | JQ008532 | **HQ993075** | **HQ913903** | JQ007903 |
| orangea1 | ZSM 1565/2008  (FGZC 1703*) | Baie des Sakalava  (ca. 5km SE Ramena), 25m | JQ008060 | JQ008216 | JQ008536 | **HQ993076** | **HQ913904** | **N/A** |
| orangea3 | ZSM 1566/2008  (FGZC 1743) | Baie des Sakalava  (ca. 5km SE Ramena), 25m | JQ008062 | JQ008218 | JQ008538 | JQ008371 | JQ008390 | JQ007908 |
| orangea4 | ZSM 1567/2008  (FGZC 1744) | Baie des Sakalava  (ca. 5km SE Ramena), 25m | JQ008063 | JQ008219 | JQ008539 | JQ008372 | JQ008391 | JQ007909 |
| orangea5 | ---  (FGZC 1765) | Baie des Sakalava  (ca. 5km SE Ramena), 25m | JQ008064 | JQ008220 | JQ008540 | JQ008373 | JQ008392 | JQ007910 |
| orangea7 | ZSM 1568/2008  (FGZC 1767) | Baie des Sakalava  (ca. 5km SE Ramena), 25m | JQ008065 | JQ008221 | JQ008541 | JQ008374 | JQ008393 | JQ007911 |
| orangea9 | ---  (FGZC 1839) | Baie des Sakalava  (ca. 5km SE Ramena), 25m | JQ008066 | JQ008222 | JQ008542 | JQ008375 | JQ008394 | JQ007912 |
| orangea12 | ---  (FGZC 1845) | Baie des Sakalava  (ca. 5km SE Ramena), 25m | JQ008061 | JQ008217 | JQ008537 | JQ008370 | JQ008389 | JQ007907 |
| amtsiranana1 | ---  (FGZC 1029) | Baie des Dunes  (E Ramena), 14m | JQ008057 | JQ008213 | JQ008533 | JQ008368 | JQ008387 | JQ007904 |
| amtsiranana2 | ---  (FGZC 1030) | Baie des Dunes  (E Ramena), 14m | JQ008058 | JQ008214 | JQ008534 | JQ008369 | JQ008388 | JQ007905 |
| amtsiranana3 | ZSM 2076/2007  (FGZC 1031) | Baie des Dunes  (E Ramena), 14m | JQ008059 | JQ008215 | JQ008535 | **HQ993074** | **HQ913902** | JQ007906 |
|  |  |  |  |  |  |  |  |  |
| ***M. igneocaudatus***  ***central clade*** | (n=8) |  |  |  |  |  |  |  |
| itremo1 | ZSM 521/2001  (2001-D35) | Itremo, 20°36'08'' 46°34'16'', 1648 m | JQ008070 | JQ008226 | JQ008546 | JQ008379 | JQ008398 | JQ007916 |
| ibity2 | ---  (2001-D15) | Mont Ibity | JQ008071 | JQ008227 | JQ008547 | JQ008380 | JQ008399 | JQ007917 |
| ibity3 | ---  (2001-D14) | Mont Ibity | JQ008072 | **FJ667677** | JQ008548 | JQ008381 | JQ008400 | JQ007918 |
| ***southern clade*** |  |  |  |  |  |  |  |  |
| Ifaty1 | XXX  (MVZC 12819) | Ifaty forest  23°07'35.8''S 43°38'02''E | JQ008075 | JQ008230 | JQ008551 | JQ008384 | JQ008403 | JQ007921 |
| ifaty2 | ZSM 1602/2010  (ZCMV13012) | Ifaty Mangily Reserve,  23° 07' 22.05''43°36'34.02'' | JQ008211 | JQ008367 | JQ008685 | JQ008386 | JQ008531 | JQ008055 |
| sakabera | XXX  (MVZC 12826) | Sakabera  23°18'11''S 43°39'31''E | JQ008076 | JQ008231 | JQ008552 | JQ008385 | JQ008404 | JQ007922 |
| anakao | ZSM 1600/2010  (MVZC 12888) | Anakao dunes  23°39'19''S 43°39'00''E | JQ008073 | JQ008228 | JQ008549 | JQ008382 | JQ008401 | JQ007919 |
| fauxcap | ZSM 1601/2010  (MVZC 12897) | Faux-Cap dunes  25°34'07''S 45°31'52''E | JQ008074 | JQ008229 | JQ008550 | JQ008383 | JQ008402 | JQ007920 |
|  |  |  |  |  |  |  |  |  |
| *« M. polleni » complex*  *northern clade* | (n=30) |  |  |  |  |  |  |  |
| amkarana1 | ZSM 1562/2008  (FGZC 1658) | Ankarana,  near Petit Tsingy, 90m | JQ008161 | JQ008317 | JQ008635 | JQ008769 | JQ008488 | JQ008007 |
| amkarana2 | ZSM 1563/2008  (FGZC 1827) | Ankarana,  near Petit Tsingy, 90m | JQ008162 | JQ008318 | JQ008636 | **HQ993081** | **HQ913909** | JQ008008 |
| ampobofofo1 | ZSM 1570/2008  (FGZC 1917) | Ampombofofo-region  (trapsite 5), 28m | JQ008163 | JQ008319 | JQ008637 | **HQ993077** | **HQ913905** | JQ008009 |
| montagnefrancais1 | ---  (FGZC 1677) | Montagne des Français,  pitfall lines 1 and 5 | JQ008175 | JQ008331 | JQ008649 | JQ008781 | JQ008500 | JQ008021 |
| montagnefrancais2 | ZSM 1574/2008  (FGZC 1678) | Montagne des Français,  pitfall lines 1 and 5 | JQ008176 | JQ008332 | JQ008650 | JQ008782 | JQ008501 | JQ008022 |
| montagnefrancais3 | 1573/2008  (FGZC 1680) | Montagne des Francais,  pitfall lines 1 and 5 | JQ008177 | JQ008333 | JQ008651 | JQ008783 | JQ008502 | JQ008023 |
| montagnefrancais4 | ---  (FGZC 1684) | Montagne des Français,  pitfall lines 1 and 5 | JQ008178 | JQ008334 | JQ008652 | JQ008784 | JQ008503 | JQ008024 |
| montagnefrancais5 | ---  (FGZC 1685) | Montagne des Français,  pitfall lines 1 and 5 | JQ008179 | JQ008335 | JQ008653 | JQ008785 | JQ008504 | JQ008025 |
| montagnefrancais6 | ZSM 1577/2008  (FGZC 1687) | Montagne des Francais,  pitfall lines 1 and 5 | JQ008180 | JQ008336 | JQ008654 | JQ008786 | JQ008505 | JQ008026 |
| montagnefrancais7 | ZSM 242/2004  (FGZC 0474) | Montagne des Francais, 334 m, 12°19‘34“S, 49°20‘09’‘E | JQ008181 | JQ008337 | JQ008655 | **HQ993078** | **HQ913906** | JQ008027 |
| montagnefrancais8 | ZSM 245/2004  (FGZC 0480) | Montagne des Francais, 334 m,  12°19‘34’‘S, 49°20‘09’‘E | JQ008182 | JQ008338 | JQ008656 | JQ008787 | JQ008506 | JQ008028 |
| montagnefrancais9 | ---  (FGZC 0481) | Montagne des Francais, 334 m, 12°19‘34’‘S, 49°20‘09’‘E | JQ008183 | JQ008339 | JQ008657 | JQ008788 | JQ008507 | JQ008029 |
| montagnefr1 | ---  (FGZC 1762) | Montagne des Francais | JQ008168 | JQ008324 | JQ008642 | JQ008774 | JQ008493 | JQ008014 |
| montagnefr2 | ---  (FGZC 1763) | Montagne des Francais | JQ008171 | JQ008327 | JQ008645 | JQ008777 | JQ008496 | JQ008017 |
| montagnefr5 | ---  (FGZC 1788) | Montagne des Francais | JQ008172 | JQ008328 | JQ008646 | JQ008778 | JQ008497 | JQ008018 |
| montagnefr6 | ---  (FGZC 1789) | Montagne des Francais | JQ008173 | JQ008329 | JQ008647 | JQ008779 | JQ008498 | JQ008019 |
| montagnefr9 | ---  (FGZC 1835) | Montagne des Français,  pitfall lines 1 and 5 | JQ008174 | JQ008330 | JQ008648 | JQ008780 | JQ008499 | JQ008020 |
| montagnefr10 | ZSM 1575/2008  (FGZC 1836) | Montagne des Francais,  pitfall lines 1 and 5 | JQ008169 | JQ008325 | JQ008643 | JQ008775 | JQ008494 | JQ008015 |
| montagnefr11 | ZSM 1576/2008  (FGZC 1838) | Montagne des Francais,  pitfall lines 1 and 5 | JQ008170 | JQ008326 | JQ008644 | JQ008776 | JQ008495 | JQ008016 |
| orangea2 | ---  (FGZC 1742) | Orangea ? | JQ008186 | JQ008342 | JQ008660 | JQ008790 | JQ008509 | JQ008031 |
| orangea6 | ZSM 1571/2008  (FGZC 1766) | Baie des Sakalava  (ca. 5km SE Ramena), 28 m | JQ008187 | JQ008343 | JQ008661 | JQ008791 | JQ008510 | JQ008032 |
| orangea8 | ---  (FGZC 1768) | Baie des Sakalava  (ca. 5km SE Ramena), 28 m | JQ008188 | JQ008344 | JQ008662 | JQ008792 | JQ008511 | JQ008033 |
| orangea10 | ---  (FGZC 1840) | Baie des Sakalava  (ca. 5km SE Ramena), 28 m | JQ008184 | JQ008340 | JQ008658 | JQ008789 | JQ008508 | JQ008030 |
| orangea11 | ZSM 1572/2008  (FGZC 1844) | Baie des Sakalava  (ca. 5km SE Ramena), 28 m | JQ008185 | JQ008341 | JQ008659 | **HQ993079** | **HQ913907** | **N/A** |
| *southern clade* |  |  |  |  |  |  |  |  |
| amkarafantsika1 | ---  (2001-B55) | Ankarafantsika | **FJ667649** | JQ008315 | JQ008633 | JQ008768 | JQ008487 | JQ008005 |
| amkarafantsika2 | ZSM 522/2001  (MV2001-313 /2001-C19) | Ankarafantsika (Ampijoroa) | JQ008160 | JQ008316 | JQ008634 | **HQ993080** | **HQ913908** | JQ008006 |
| kirindy1 | ZSM 1596/2010  (MVZC 12755) | Kirindy, pitfall line  20°04’40.0’’S, 44°41’30.1’’E | JQ008164 | JQ008320 | JQ008638 | JQ008770 | JQ008489 | JQ008010 |
| kirindy2 | ZSM 1599/2010  (MVZC 12914) | Kirindy, pitfall line  20°04’40.0’’S, 44°41’30.1’’E | JQ008165 | JQ008321 | JQ008639 | JQ008771 | JQ008490 | JQ008011 |
| kirindy3 | ZSM 1598/2010  (MVZC 12915) | Kirindy, pitfall line  20°04’40.0’’S, 44°41’30.1’’E | JQ008166 | JQ008322 | JQ008640 | JQ008772 | JQ008491 | JQ008012 |
| kirindy4 | ZSM 1597/2010  (MVZC 12916) | Kirindy, pitfall line  20°04’40.0’’S, 44°41’30.1’’E | JQ008167 | JQ008323 | JQ008641 | JQ008773 | JQ008492 | JQ008013 |
| ***M. stumpffi*** | (n=22) |  |  |  |  |  |  |  |
|  |  |  |  |  |  |  |  |  |
| ambre1 | ZSM 1558/2008  (FGZC 3124) | Forêt d'Ambre ca. 5km SW Sakaramy (trapside), 479 m | JQ008189 | JQ008345 | JQ008663 | JQ008793 | JQ008512 | JQ008034 |
| ambre2 | ---  (FGZC 3123) | Forêt d'Ambre ca. 5km SW Sakaramy (trapside), 479 m | JQ008190 | JQ008346 | JQ008664 | JQ008794 | JQ008513 | JQ008035 |
| antanambao | ZSM 206/2003  (---) | Antanambao (Maevatanana, on way to Manongarivo) | JQ008191 | JQ008347 | JQ008665 | **HQ993083** | **HQ913911** | JQ008036 |
| antsirasira1 | ---  (2001-G44) | Antsirasira | JQ008192 | JQ008348 | JQ008666 | JQ008795 | JQ008514 | JQ008037 |
| marojejy1 | ---  (ZCMV 2026) | Marojejy, 481m asl  14°26.260´S, 49°46.533’E | JQ008193 | JQ008349 | JQ008667 | JQ008796 | JQ008515 | JQ008038 |
| marojejy2 | ZSM 0032/2005  (ZCMV 2031) | Marojejy, 481m asl  14°26.260´S, 49°46.533’E | JQ008196 | JQ008352 | JQ008670 | **HQ993084** | **HQ913912** | JQ008041 |
| marojejy3 | 0033/2005  (ZCMV 2032) | Marojejy, 481m asl  14°26.260´S, 49°46.533’E | JQ008197 | JQ008353 | JQ008671 | JQ008799 | JQ008518 | JQ008042 |
| marojejy4 | 0404/2005  (ZCMV 2033) | Marojejy, 481m asl  14°26.260´S, 49°46.533’E | JQ008198 | JQ008354 | JQ008672 | JQ008800 | JQ008519 | JQ008043 |
| marojejy5 | 0405/2005  (ZCMV 2034) | Marojejy, 481m asl  14°26.260´S, 49°46.533’E | JQ008199 | JQ008355 | JQ008673 | JQ008801 | JQ008520 | JQ008044 |
| marojejy6 | 0040/2005  (FGZC 2784) | Marojejy, 481m asl  14°26.260´S, 49°46.533’E | JQ008200 | JQ008356 | JQ008674 | JQ008802 | JQ008521 | JQ008045 |
| marojejy7 | 0041/2005  (FGZC 2785) | Marojejy, 481m asl  14°26.260´S, 49°46.533’E | JQ008201 | JQ008357 | JQ008675 | JQ008803 | JQ008522 | JQ008046 |
| marojejy8 | ---  (FGZC 2786) | Marojejy, 481m asl  14°26.260´S, 49°46.533’E | JQ008202 | JQ008358 | JQ008676 | JQ008804 | JQ008523 | JQ008047 |
| marojejy9 | 0043/2005  (FGZC 2787) | Marojejy, 481m asl  14°26.260´S, 49°46.533’E | JQ008203 | JQ008359 | JQ008677 | JQ008805 | JQ008524 | JQ008048 |
| marojejy10 | ---  (FGZC 2788) | Marojejy, 481m asl  14°26.260´S, 49°46.533’E | JQ008194 | JQ008350 | JQ008668 | JQ008797 | JQ008516 | JQ008039 |
| marojejy11 | ---  (FGZC 2789) | Marojejy, 481m asl  14°26.260´S, 49°46.533’E | JQ008195 | JQ008351 | JQ008669 | JQ008798 | JQ008517 | JQ008040 |
| mtambre | ZSM 2162/2007  (FGZC1228) | Forêt d'Ambre ca. 4km WSW Sakaramy, 470m | JQ008210 | JQ008366 | JQ008684 | JQ008811 | JQ008530 | JQ008054 |
| montagnefr3 | ---  (FGZC 1764) | Montagne des Francais | JQ008206 | JQ008362 | JQ008680 | JQ008808 | JQ008527 | JQ008050 |
| montagnefr4 | ZSM 1560/2008  (FGZC 1772) | Montagne des Français, (pitfalls) | JQ008207 | JQ008363 | JQ008681 | JQ008809 | JQ008528 | JQ008051 |
| montagnefr7 | ---  (FGZC 1822) | Montagne des Français, (pitfalls) | JQ008208 | JQ008364 | JQ008682 | JQ008810 | JQ008529 | JQ008052 |
| montagnefr8 | ZSM 1561/2008  (FGZC 1823) | Montagne des Français, (pitfalls) | JQ008209 | JQ008365 | JQ008683 | **HQ993082** | **HQ913910** | JQ008053 |
| montagnefr12 | ---  (FGZC 1722) | Montagne des Francais | JQ008204 | JQ008360 | JQ008678 | JQ008806 | JQ008525 | JQ008049 |
| montagnefr13 | ---  (FGZC 1723) | Montagne des Francais | JQ008205 | JQ008361 | JQ008679 | JQ008807 | JQ008526 | **N/A** |
| *M. nanus* | (n=3) |  |  |  |  |  |  |  |
| andasibe1 | ---  (ZCMV 2283) | Andasibe | JQ008067 | JQ008223 | JQ008543 | JQ008376 | JQ008395 | JQ007913 |
| andasibe2 | ---  (ZCMV 2284) | Andasibe | JQ008068 | JQ008224 | JQ008544 | JQ008377 | JQ008396 | JQ007914 |
| andasibe3 | ---  (ZCMV 2285) | Andasibe | JQ008069 | JQ008225 | JQ008545 | JQ008378 | JQ008397 | JQ007915 |
|  |  |  |  |  |  |  |  |  |
| *M. mouroundavae* | (n=6) |  |  |  |  |  |  |  |
| andasibe1 | ---  (ZCMV 2252) | Andasibe (pitfall), 939 m  18°56.169'S, 48°24.734'E | JQ008138 | JQ008293 | JQ008611 | JQ008746 | JQ008465 | JQ007984 |
| andasibe2 | ZSM 12/2005  (ZCMV 2253) | Andasibe (pitfall), 939 m  18°56.169'S, 48°24.734'E | JQ008139 | JQ008294 | JQ008612 | JQ008747 | JQ008466 | JQ007985 |
| andasibe3 | ZSM 13/2005  (ZCMV 2254) | Andasibe (pitfall), 939 m  18°56.169'S, 48°24.734'E | JQ008140 | JQ008295 | JQ008613 | JQ008748 | JQ008467 | JQ007986 |
| andasibe4 | ZSM 31/2005  (ZCMV 2289) | Andasibe (pitfall), 939 m  18°56.169'S, 48°24.734'E | JQ008141 | JQ008296 | JQ008614 | JQ008749 | JQ008468 | JQ007987 |
| andasibe5 | ZSM 6/2005  (ZCMV 2239) | Andasibe (pitfall), 939 m  18°56.169'S, 48°24.734'E | JQ008142 | JQ008297 | JQ008615 | JQ008750 | JQ008469 | JQ007988 |
| ambre | ---  (ACZC 1635) | near Montagne d’Ambre  12°35’08.1’’S, 49° 08’44.5’’E | JQ008143 | JQ008298 | JQ008616 | JQ008751 | JQ008470 | JQ007989 |
|  |  |  |  |  |  |  |  |  |
| *« M. melanopleura »* | (n=77) |  |  |  |  |  |  |  |
| *central clade* |  |  |  |  |  |  |  |  |
| anala2 | ---  (ZCMV 2404) | An'Ala forest camp, 889 m  18.91926°S, 48.48796°E | JQ008081 | JQ008236 | **N/A** | JQ008690 | JQ008409 | JQ007927 |
| andasibe1 | UADBA collection (ZCMV 2257) | Andasibe | JQ008082 | JQ008237 | JQ008557 | **HQ993073** | **HQ913901** | JQ007928 |
| andasibe2 | ZSM 14/2005  (ZCMV 2258) | Andasibe pitfall, , 939 m  18°56.169'S, 48°24.734'E | JQ008093 | JQ008248 | JQ008567 | JQ008701 | JQ008420 | JQ007939 |
| andasibe3 | ---  (ZCMV 2259) | Andasibe pitfall | JQ008104 | JQ008259 | JQ008578 | JQ008712 | JQ008431 | JQ007950 |
| andasibe4 | ZSM 15/2005  (ZCMV 2260) | Andasibe pitfall, , 939 m  18°56.169'S, 48°24.734'E | JQ008107 | JQ008262 | JQ008581 | JQ008715 | JQ008434 | JQ007953 |
| andasibe5 | ---  (ZCMV 2261) | Andasibe pitfall | JQ008108 | JQ008263 | JQ008582 | JQ008716 | JQ008435 | JQ007954 |
| andasibe6 | ZSM 16/2005  (ZCMV 2262) | Andasibe pitfall, , 939 m  18°56.169'S, 48°24.734'E | JQ008109 | JQ008264 | JQ008583 | JQ008717 | JQ008436 | JQ007955 |
| andasibe7 | ZSM 17/2005  (ZCMV 2263) | Andasibe pitfall, , 939 m  18°56.169'S, 48°24.734'E | JQ008110 | JQ008265 | JQ008584 | JQ008718 | JQ008437 | JQ007956 |
| andasibe8 | ZSM 18/2005  (ZCMV 2264) | Andasibe pitfall, , 939 m  18°56.169'S, 48°24.734'E | JQ008111 | JQ008266 | JQ008585 | JQ008719 | JQ008438 | JQ007957 |
| andasibe9 | ZSM 19/2005  (ZCMV 2265) | Andasibe pitfall, , 939 m  18°56.169'S, 48°24.734'E | JQ008112 | JQ008267 | JQ008586 | JQ008720 | JQ008439 | JQ007958 |
| andasibe10 | ZSM 20/2005  (ZCMV 2266) | Andasibe pitfall, , 939 m  18°56.169'S, 48°24.734'E | JQ008083 | JQ008238 | JQ008558 | JQ008691 | JQ008410 | JQ007929 |
| andasibe11 | ZSM 21/2005  (ZCMV 2267) | Andasibe pitfall, , 939 m  18°56.169'S, 48°24.734'E | JQ008084 | JQ008239 | JQ008559 | JQ008692 | JQ008411 | JQ007930 |
| andasibe12 | ZSM 22/2005  (ZCMV 2268) | Andasibe pitfall, , 939 m  18°56.169'S, 48°24.734'E | JQ008085 | JQ008240 | JQ008560 | JQ008693 | JQ008412 | JQ007931 |
| andasibe13 | ZSM 23/2005  (ZCMV 2269) | Andasibe pitfall, , 939 m  18°56.169'S, 48°24.734'E | JQ008086 | JQ008241 | JQ008561 | JQ008694 | JQ008413 | JQ007932 |
| andasibe14 | ---  (ZCMV 2270) | Andasibe pitfall | JQ008087 | JQ008242 | **N/A** | JQ008695 | JQ008414 | JQ007933 |
| andasibe15 | ZSM 24/2005  (ZCMV 2271) | Andasibe pitfall, | JQ008088 | JQ008243 | JQ008562 | JQ008696 | JQ008415 | JQ007934 |
| andasibe16 | ---  (ZCMV 2272) | Andasibe pitfall, , 939 m  18°56.169'S, 48°24.734'E | JQ008089 | JQ008244 | JQ008563 | JQ008697 | JQ008416 | JQ007935 |
| andasibe17 | ZSM 25/2005  (ZCMV 2273) | Andasibe pitfall, , 939 m  18°56.169'S, 48°24.734'E | JQ008090 | JQ008245 | JQ008564 | JQ008698 | JQ008417 | JQ007936 |
| andasibe18 | ZSM 26/2005  (ZCMV 2274) | Andasibe pitfall, , 939 m  18°56.169'S, 48°24.734'E | JQ008091 | JQ008246 | JQ008565 | JQ008699 | JQ008418 | JQ007937 |
| andasibe19 | ---  (ZCMV 2275) | Andasibe pitfall | JQ008092 | JQ008247 | JQ008566 | JQ008700 | JQ008419 | JQ007938 |
| andasibe20 | ---  (ZCMV 2276) | Andasibe pitfall | JQ008094 | JQ008249 | JQ008568 | JQ008702 | JQ008421 | JQ007940 |
| andasibe21 | ---  (ZCMV 2277) | Andasibe pitfall | JQ008095 | JQ008250 | JQ008569 | JQ008703 | JQ008422 | JQ007941 |
| andasibe22 | ---  (ZCMV 2278) | Andasibe pitfall | JQ008096 | JQ008251 | JQ008570 | JQ008704 | JQ008423 | JQ007942 |
| andasibe23 | ZSM 27/2005  (ZCMV 2279) | Andasibe pitfall, , 939 m  18°56.169'S, 48°24.734'E | JQ008097 | JQ008252 | JQ008571 | JQ008705 | JQ008424 | JQ007943 |
| andasibe24 | ZSM 28/2005  (ZCMV 2280) | Andasibe pitfall, , 939 m  18°56.169'S, 48°24.734'E | JQ008098 | JQ008253 | JQ008572 | JQ008706 | JQ008425 | JQ007944 |
| andasibe25 | ---  (ZCMV 2281) | Andasibe pitfall | JQ008099 | JQ008254 | JQ008573 | JQ008707 | JQ008426 | JQ007945 |
| andasibe26 | ---  (ZCMV 2282) | Andasibe pitfall | JQ008100 | JQ008255 | JQ008574 | JQ008708 | JQ008427 | JQ007946 |
| andasibe27 | ZSM 2/2005  (ZCMV 2232) | Andasibe pitfall, , 939 m  18°56.169'S, 48°24.734'E | JQ008101 | JQ008256 | JQ008575 | JQ008709 | JQ008428 | JQ007947 |
| andasibe28 | ZSM 3/2005  (ZCMV 2233) | Andasibe pitfall, , 939 m  18°56.169'S, 48°24.734'E | JQ008102 | JQ008257 | JQ008576 | JQ008710 | JQ008429 | JQ007948 |
| andasibe29 | ZSM 4/2005  (ZCMV 2234) | Andasibe pitfall, , 939 m  18°56.169'S, 48°24.734'E | JQ008103 | JQ008258 | JQ008577 | JQ008711 | JQ008430 | JQ007949 |
| andasibe30 | ---  (ZCMV 2235) | Andasibe pitfall | JQ008105 | JQ008260 | JQ008579 | JQ008713 | JQ008432 | JQ007951 |
| andasibe31 | ---  (ZCMV 2236) | Andasibe pitfall | JQ008106 | JQ008261 | JQ008580 | JQ008714 | JQ008433 | JQ007952 |
| andasibe-x1 | ZSM 334/2004  (FGZC 631) | Andasibé (?) | JQ008157 | JQ008312 | JQ008630 | JQ008765 | JQ008484 | JQ008002 |
| andasibe-x2 | ZSM 335/2004  (FGZC 632) | Andasibé (?) | JQ008158 | JQ008313 | JQ008631 | JQ008766 | JQ008485 | JQ008003 |
| andasibe-x3 | ZSM 333/2004  (FGZC 630) | Andasibé (?) | JQ008159 | JQ008314 | JQ008632 | JQ008767 | JQ008486 | JQ008004 |
| ambohitsara1 | ---  (ZCMV 0081) | Ambohitsara | JQ008079 | JQ008234 | JQ008555 | JQ008688 | JQ008407 | JQ007925 |
| fierenana1 | ---  (2002-2508) | Fierenana | JQ008117 | JQ008272 | JQ008591 | JQ008725 | JQ008444 | JQ007963 |
| fierenana2 | ---  (2002-2351) | Fierenana | JQ008118 | JQ008273 | JQ008592 | JQ008726 | JQ008445 | JQ007964 |
| mahasoa | ---  (DRV 5632) | Mahasoa | JQ008124 | JQ008279 | **N/A** | JQ008732 | JQ008451 | JQ007970 |
| sahafina2 | ---  (PSG 2008-291) | Sahafina forest,  near Brickaville | JQ008152 | JQ008307 | JQ008625 | JQ008760 | JQ008479 | JQ007998 |
| sahafina3 | ---  (PSG 2008-290) | Sahafina forest,  near Brickaville | JQ008153 | JQ008308 | JQ008626 | JQ008761 | JQ008480 | JQ007999 |
| torotorofotsy1 | ---  (ZCMV 812) | Torotorofotsy | JQ008154 | JQ008309 | JQ008627 | JQ008762 | JQ008481 | **N/A** |
|  |  |  |  |  |  |  |  |  |
| *southern clade* |  |  |  |  |  |  |  |  |
| ambatolahy1 | ZSM 354/2006  (ZCMV 3061) | Ambatolahy river, 915 m  21°14.632'S, 47°25.573'E | JQ008077 | JQ008232 | JQ008553 | JQ008686 | JQ008405 | JQ007923 |
| ambatolahy2 | ---  (ZCMV 608) | Ambatolahy forest | JQ008078 | JQ008233 | JQ008554 | JQ008687 | JQ008406 | JQ007924 |
| andohahela | ---  (FGZC 0204) | Andohahela | JQ008113 | JQ008268 | JQ008587 | JQ008721 | JQ008440 | JQ007959 |
| imaloka1 | ---  (ZCMV 2957) | Imaloka | JQ008119 | JQ008274 | JQ008593 | JQ008727 | JQ008446 | JQ007965 |
| imaloka2 | ---  (ZCMV 2958) | Imaloka | JQ008120 | JQ008275 | JQ008594 | JQ008728 | JQ008447 | JQ007966 |
| imaloka3 | ---  (ZCMV 2959) | Imaloka | JQ008121 | JQ008276 | JQ008595 | JQ008729 | JQ008448 | JQ007967 |
| imaloka4 | ---  (ZCMV 2960) | Imaloka | JQ008122 | JQ008277 | JQ008596 | JQ008730 | JQ008449 | JQ007968 |
| imaloka5 | ZSM 356/2006  (ZCMV 2961) | Imaloka, 1020 m,  21°14.527'S, 47°27.909'E | JQ008123 | JQ008278 | JQ008597 | JQ008731 | JQ008450 | JQ007969 |
| ranomafana1 | ---  (ZCMV 2910) | Ranomafana Park | JQ008144 | JQ008299 | JQ008617 | JQ008752 | JQ008471 | JQ007990 |
| ranomafana2 | ---  (ZCMV 0032) | Ranomafana, Kidonavo bridge | JQ008145 | JQ008300 | JQ008618 | JQ008753 | JQ008472 | JQ007991 |
| ranomafana3 | ---  (ZCMV 147) | Ranomafana, Kidonavo bridge | JQ008146 | JQ008301 | JQ008619 | JQ008754 | JQ008473 | JQ007992 |
| ranomafana5 | ---  (ZCMV 148) | Ranomafana, Kidonavo bridge | JQ008147 | JQ008302 | JQ008620 | JQ008755 | JQ008474 | JQ007993 |
| ranomafanakely1 | ZSM 355/2006  (ZCMV 2907) | Ranomafanakely, 1134 m  21°14.921'S, 47°22.307'E | JQ008148 | JQ008303 | JQ008621 | JQ008756 | JQ008475 | JQ007994 |
| ranomafanakely2 | ---  (ZCMV 2955) | Ranomafanakely, pitfall site | JQ008149 | JQ008304 | JQ008622 | JQ008757 | JQ008476 | JQ007995 |
| ranomafanakely3 | ---  (ZCMV 3064) | Ranomafanakely pitfall | JQ008150 | JQ008305 | JQ008623 | JQ008758 | JQ008477 | JQ007996 |
| ranomafanakely4 | ---  (ZCMV 3065) | Ranomafanakely pitfall | JQ008151 | JQ008306 | JQ008624 | JQ008759 | JQ008478 | JQ007997 |
|  |  |  |  |  |  |  |  |  |
| *northern clade* |  |  |  |  |  |  |  |  |
| anala1 | ZSM 207/2006  (ZCMV 2481) | An'Ala forest camp, 889 m  18.91926°S, 48.48796°E | JQ008080 | JQ008235 | JQ008556 | JQ008689 | JQ008408 | JQ007926 |
| angozongahy1 | ---  (ZCMV 11209) | Angozongahy (camp 1) | JQ008114 | JQ008269 | JQ008588 | JQ008722 | JQ008441 | JQ007960 |
| mtambre1 | ---  (ACZC 1686) | near Montagne d’Ambre  12°34’52.6’’S, 49°08’24.0’’E | JQ008115 | JQ008270 | JQ008589 | JQ008723 | JQ008442 | JQ007961 |
| mtambre2 | ---  (ACZC 1694) | near Montagne d’Ambre  12 34 52.6 - 049 08 24.0 | JQ008116 | JQ008271 | JQ008590 | JQ008724 | JQ008443 | JQ007962 |
| nosymangabe2 | ---  (ZCMV 2162) | Nosy Mangabe | JQ008131 | JQ008286 | JQ008604 | JQ008739 | JQ008458 | JQ007977 |
| nosymangabe3 | ZSM 398/2005  (ZCMV 2163) | Nosy Mangabe, ca. 50-100 m  15°30'S, 49°46'E | JQ008132 | JQ008287 | JQ008605 | JQ008740 | JQ008459 | JQ007978 |
| nosymangabe4 | ZSM 399/2005  (ZCMV 2164) | Nosy Mangabe, ca. 50-100 m  15°30'S, 49°46'E | JQ008133 | JQ008288 | JQ008606 | JQ008741 | JQ008460 | JQ007979 |
| nosymangabe5 | ---  (ZCMV 2165) | Nosy Mangabe | JQ008134 | JQ008289 | JQ008607 | JQ008742 | JQ008461 | JQ007980 |
| nosymangabe6 | ZSM 400/2005  (ZCMV 2166) | Nosy Mangabe, ca. 50-100 m  15°30'S, 49°46'E | JQ008135 | JQ008290 | JQ008608 | JQ008743 | JQ008462 | JQ007981 |
| nosymangabe7 | ZSM 401/2005  (ZCMV 2167) | Nosy Mangabe, ca. 50-100 m  15°30'S, 49°46'E | JQ008136 | JQ008291 | JQ008609 | JQ008744 | JQ008463 | JQ007982 |
| nosymangabe8 | ---  (ZCMV 2168) | Nosy Mangabe | JQ008137 | JQ008292 | JQ008610 | JQ008745 | JQ008464 | JQ007983 |
| makira1 | ---  (DRV 5838) | Makira | JQ008125 | JQ008280 | JQ008598 | JQ008733 | JQ008452 | JQ007971 |
| makira2 | ---  (DRV 5839) | Makira | JQ008126 | JQ008281 | JQ008599 | JQ008734 | JQ008453 | JQ007972 |
| makira3 | ---  (DRV 5919) | Makira | JQ008127 | JQ008282 | JQ008600 | JQ008735 | JQ008454 | JQ007973 |
| makira4 | ---  (DRV 5936) | Makira | JQ008128 | JQ008283 | JQ008601 | JQ008736 | JQ008455 | JQ007974 |
| makira6 | ---  (DRV 5920) | Makira | JQ008129 | JQ008284 | JQ008602 | JQ008737 | JQ008456 | JQ007975 |
| makira7 | ---  (DRV 5937) | Makira | JQ008130 | JQ008285 | JQ008603 | JQ008738 | JQ008457 | JQ007976 |
| tsaratanana1 | ---  (DRV 6407) | Tsaratanana mountain | JQ008155 | JQ008310 | JQ008628 | JQ008763 | JQ008482 | JQ008000 |
| tsaratanana2 | ---  (2001G-4) | Tsaratanana mountain | JQ008156 | JQ008311 | JQ008629 | JQ008764 | JQ008483 | JQ008001 |
| **Outgroups** |  |  |  |  |  |  |  |  |
| *Paracontias fasika* | ZSM 2256/2007  (FGZC1347) | Baie de Sakalava | **FJ667661** | **FJ667690** | **FJ667719** | **FJ744589** | **FJ667748** | **FJ667632** |
| *Amphiglossus meva* | UADBA 294404  (APR 05959) | Réserve Spéciale de Marotandrano | **JF424686|** | **JF424691** | **JF424710** | **JF424702** | **JF424716** | **JF424677** |

**References :**

**Miralles A**, Kölhler J, Glaw F, Vences M (2011a) A molecular phylogeny of the *Madascincus polleni* species complex, with description of a new species of scincid lizard from the coastal dune area of northern Madagascar. Zootaxa. 2876:1–16.

**Miralles A**, Raselimanana AP, Rakotomalala D, Vences M, Vieites DR (2011b) A new large and colorful skink of the genus *Amphiglossus* from Madagascar revealed by morphology and multilocus molecular study. Zootaxa. 2918:47–67.

**Miralles A**, Köhler J, Vieites DR, Glaw F, Vences M (2011c) Developing hypotheses on rostral shield evolution in head-first digging squamates from a molecular phylogeny and new species of the genus *Paracontias* (Scincidae). Org. Diver. Evol. 11:135–150.
