# Supplementary material for: New Metrics for Comparison of Taxonomies Reveal Striking Discrepancies among Species Delimitation Methods in Madascincus Lizards
Source: PLoS One. 2013 Jul 12;8(7):e68242. doi: 10.1371/journal.pone.0068242 (PMC3710018; doi:10.1371/journal.pone.0068242)
Supplement: File S6 — Phylogenetic results: complementary analyses. Bayesian Inference analyses of phylogeny carried out under different partition schemes to understand whether these would influence the general topology of the Madascincus phylogenetic tree and thus reveal flaws in the guide tree used for the BAT, WP and GMYC approaches. a. Comparison of phylogenetic trees based on nDNA and mtDNA data. b. Bayesian Inference tree of the mtDNA data set. c. Bayesian Inference tree of the nDNA data set under a partition scheme with 1st, 2nd and 3rd codon position (merged for all nuclear genes) defined as separate partitions. d. Bayesian Inference tree of the nDNA data set under a partition scheme with 1st, 2nd and 3rd codon position (separately for all nuclear genes) defined as separate partitions. e. Species trees (cladogram view) calculated with *BEAST, based on the combined mtDNA and nDNA data (above), and the combined nDNA data only (under). f. Species trees (cladogram view) calculated with *BEAST, based on the combined nDNA data, with individuals combined to terminal taxa on the basis of the analysis with STRUCTURE. (DOC) [file pone.0068242.s006.doc]

**S6. Phylogenetic results: complementary analyses**

Bayesian Inference analyses with MrBayes 1.2, with characteristics as described above for the main analyses, were also carried out under different partition schemes to understand whether these would influence the general topology of the *Madascincus* phylogenetic tree and thus reveal flaws in the guide tree used for the BAT, WP and GMYC approaches. We first performed unpartioned analyses of the mtDNA dataset and the nDNA dataset and compared them (S6a), considering that recovering congruent topologies in inferences based on independent datasets (here: unlinked molecular loci) is one of the most relevant criteria to assess clade reliability (Li & Lecointre 2009). We also partitioned the mtDNA data set keeping the (relatively short) 16S rRNA fragment as one partition and defining each codon position of ND2 as separate partition (S6b). For the nDNA dataset, we used a scheme with 3 partitions (each codon position of the combined nuclear genes defined as separate partition; S6c) and one with 12 partitions (each codon position of each nuclear gene defined as separate partition; S6d).

We furthermore used *BEAST 1.7.4 (Drummond et al. 2012) to calculate coalescence-based species trees. While this method is appropriate to reconstruct phylogenies based on multilocus datasets and in some cases might outperform concatenated datasets (e.g., Liu et al. 2009), it is strongly dependent on a correct a-priori assignment of individuals to species which then become the unit of analysis. Using such a method in a study where species delimitation is the core question therefore is risking circularity. We applied the species tree approach on one hand with species units as delimited by ITAX (species tree calculation based on combined mtDNA and nDNA data, and on nDNA data only; S6e) and on the other hand with the output of the STRUCTURE analysis as a basis (nDNA only), using each cluster as terminal unit (species), with three different alternative treatments of the ambiguously assigned specimens (S6f). Each final analysis consisted of combining six runs with MCMC chains set to 500 million generations each. Parameter files were examined in Tracer 1.5 to ascertain convergence and adequate effective sample sizes. Species tree files were combined in Tree Annotator 1.7.2 with a conservative burn-in of 50%.

**S6a.** Comparison of phylogenetic trees based on nDNA and mtDNA sequences. Nuclear DNA tree inferred from BI analysis of concatenated BDNF, CMOS, PDC and RAG2 sequences compared to the mitochondrial DNA inferred from ND1and 16S sequences (unpartitioned data-set; posterior probabilities indicated for each node).


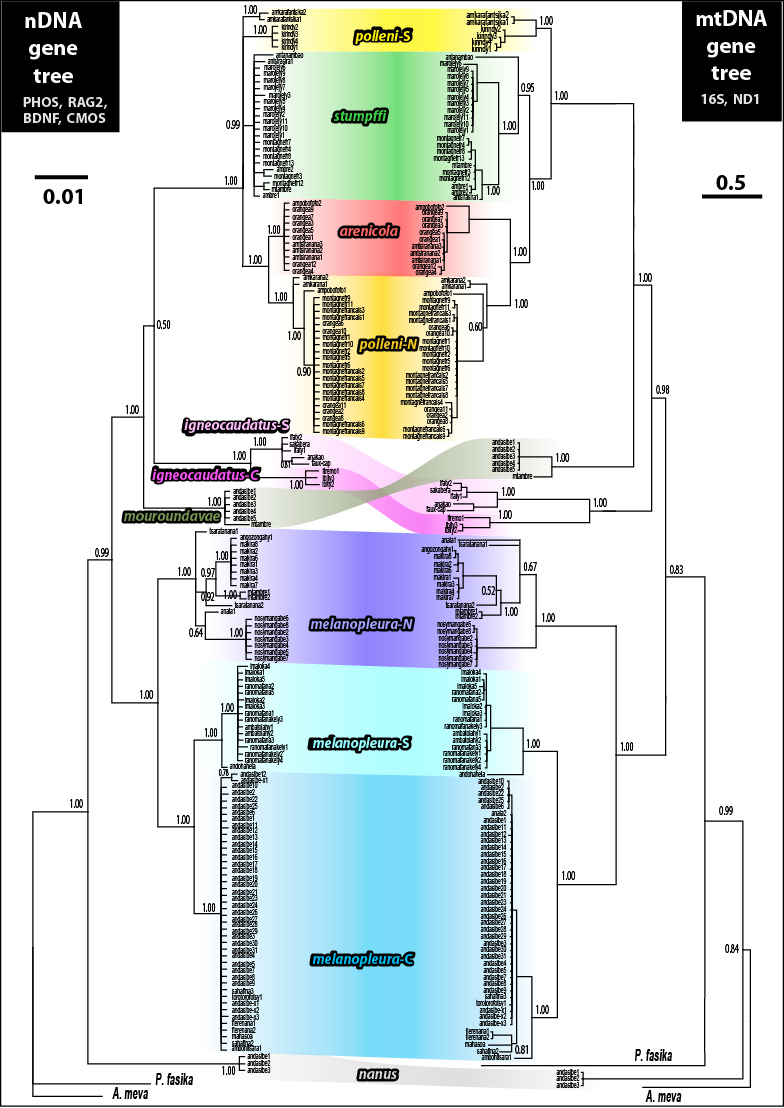


**S6b.** Bayesian Inference tree of the mtDNA data (50% majority-rule consensus, compatible nodes kept), calculated with MrBayes 1.2 under a partition scheme with each codon of ND2 and the 16SrRNA gene as separate partitions. Numbers at nodes are posterior probabilities (only values >0.9 shown).

**S6c.** Bayesian Inference tree of the nDNA data set (50% majority-rule consensus, compatible nodes kept), calculated with MrBayes 1.2 under a partition scheme with 1st, 2nd and 3rd codon position (merged for all nuclear genes) defined as separate partitions. Numbers at nodes are posterior probabilities (only values >0.9 shown).


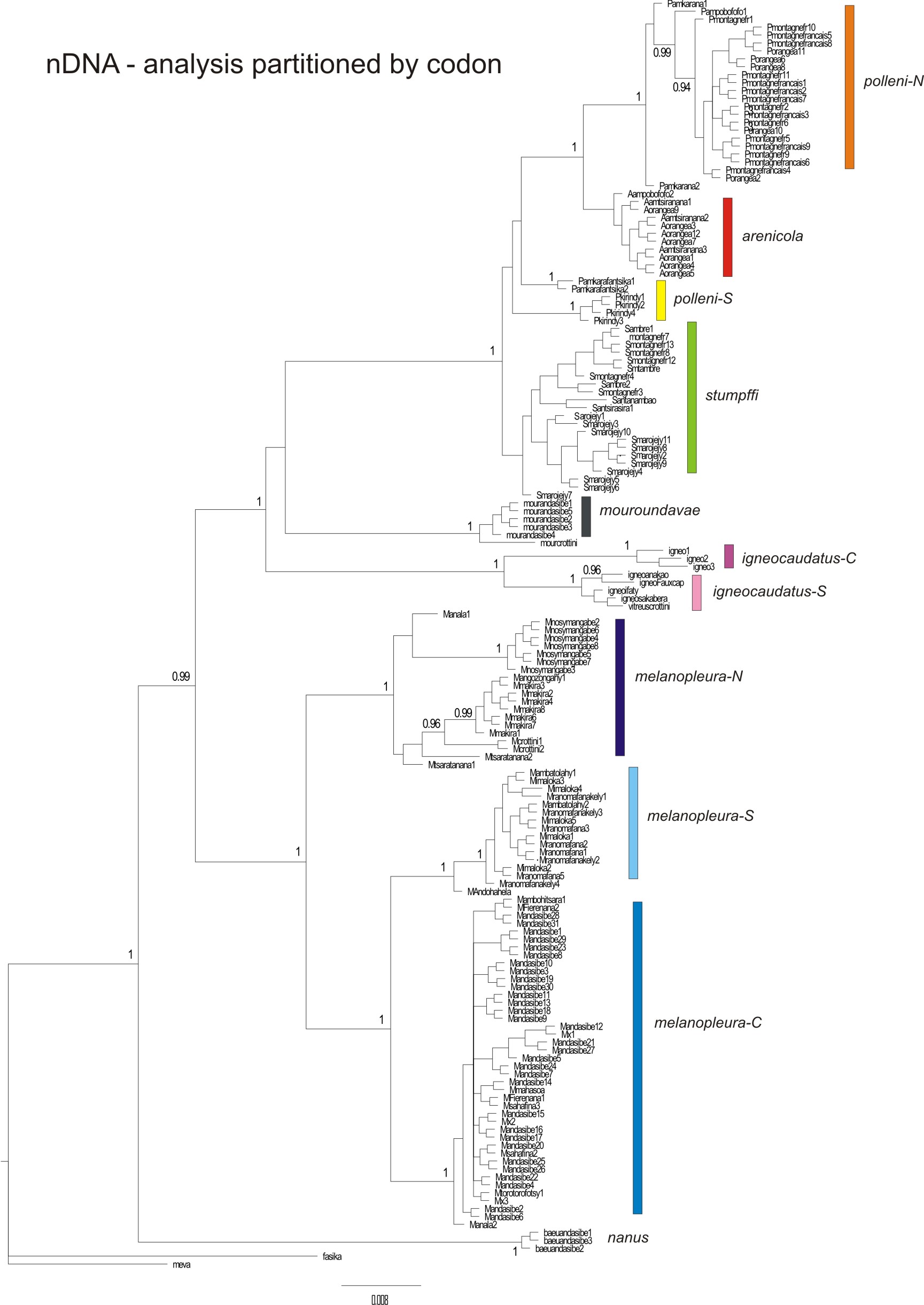


**S6d.** Bayesian Inference tree of the nDNA data set (50% majority-rule consensus, compatible nodes kept), calculated with MrBayes 1.2 under a partition scheme with 1st, 2nd and 3rd codon position (separately for all nuclear genes) defined as separate partitions (12 partitions in total). Numbers at nodes are posterior probabilities (only values >0.9 shown).

**
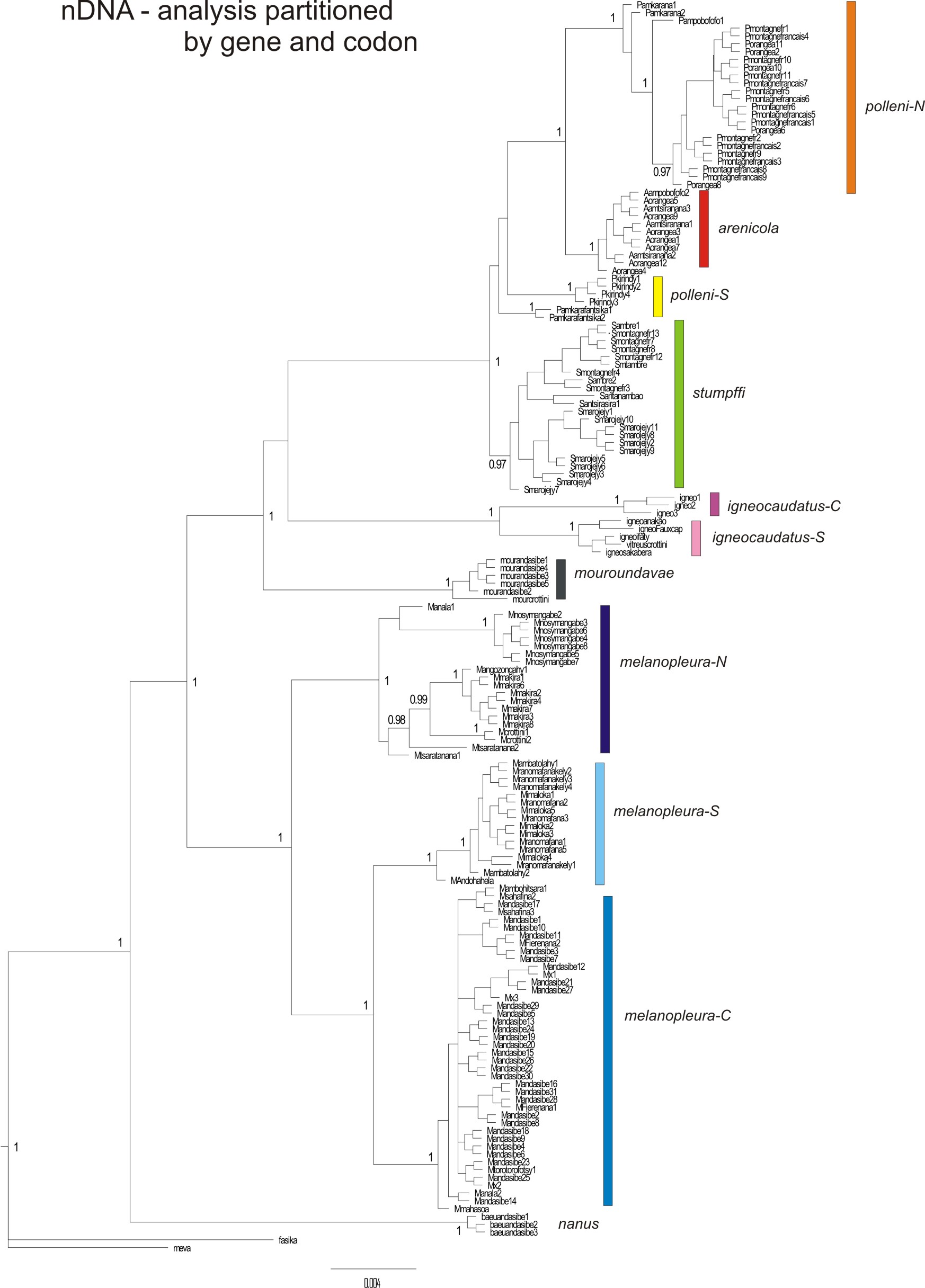
**

**S6e.** Species trees (cladogram view) calculated with *BEAST, based on the combined mtDNA and nDNA data (above), and the combined nDNA data only (under). Individuals were combined to terminal taxa on the basis of the ITAX species delimitation method results. Numbers at nodes are posterior probabilities.


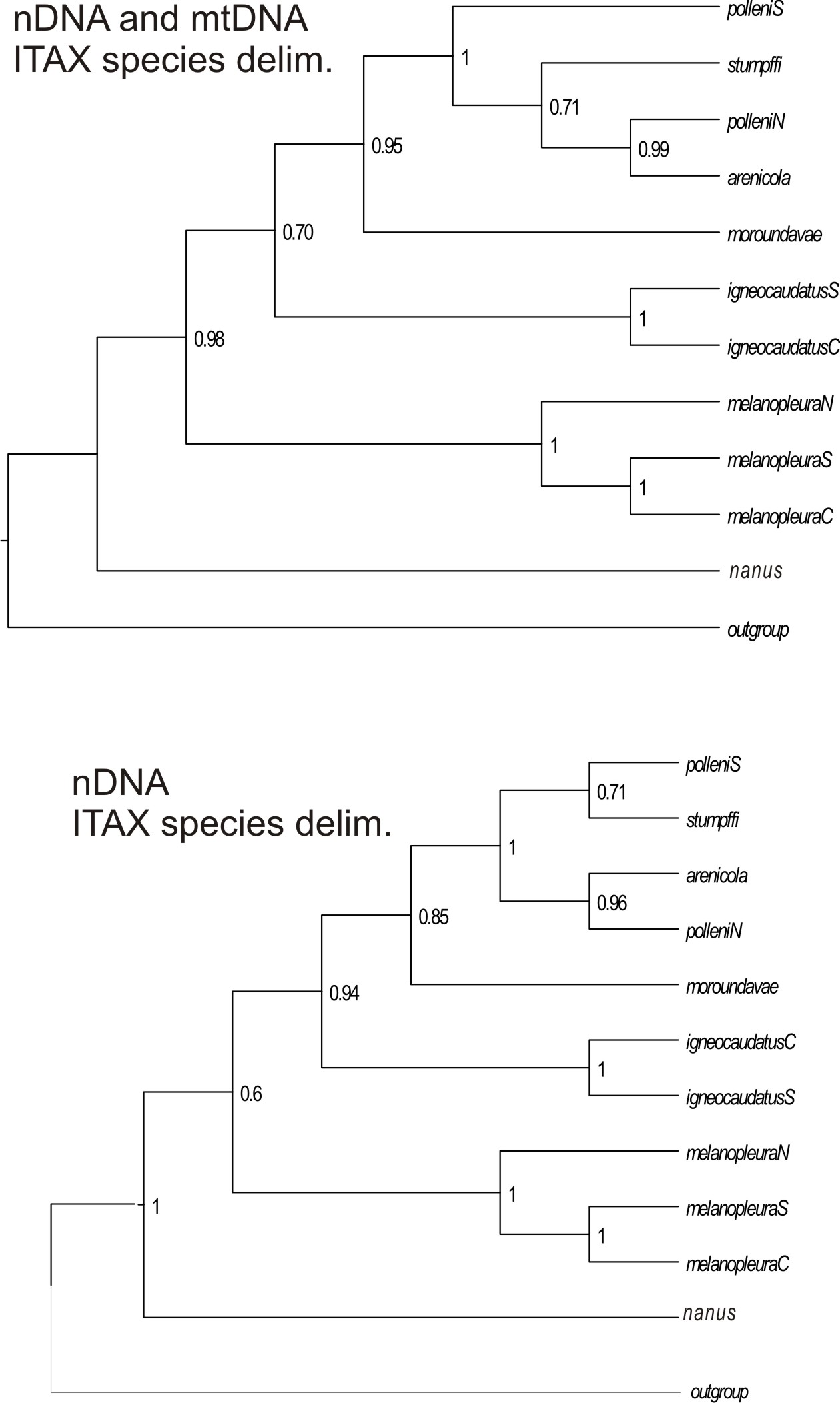


**S6f.** Species trees (cladogram view) calculated with *BEAST, based on the combined nDNA data. Individuals were combined to terminal taxa on the basis of the analysis with STRUCTURE (colored plot on the right of each tree), under three different species delimitation scenarios: Alternative 1 groups in *melanopleura-N* the populations placed in the blue cluster and those placed in the pink cluster as separate taxa, respectively, but left the morphologically very distinct species *igneocaudatus-S* (in the pink cluster as some *melanopleura-N*) and *nanus* (in the purple cluster as *igneocaudatus-C*) as separate species. Alternative 2 differed by defining the specimens of *melanopleura-N* in the pink cluster into two separate species because they formed two lineages that were not sister to each other. Alternative 3 followed strictly the STRUCTURE assignment. Note that this third tree is characterized by paraphyly of the *igneocaudatus* clade and the *melanopleura* group, and by a drop of posterior probabilities in the relationships among the taxa in these groups, in agreement with our interpretation of some aspects in the assignment test as artifacts (see S7).


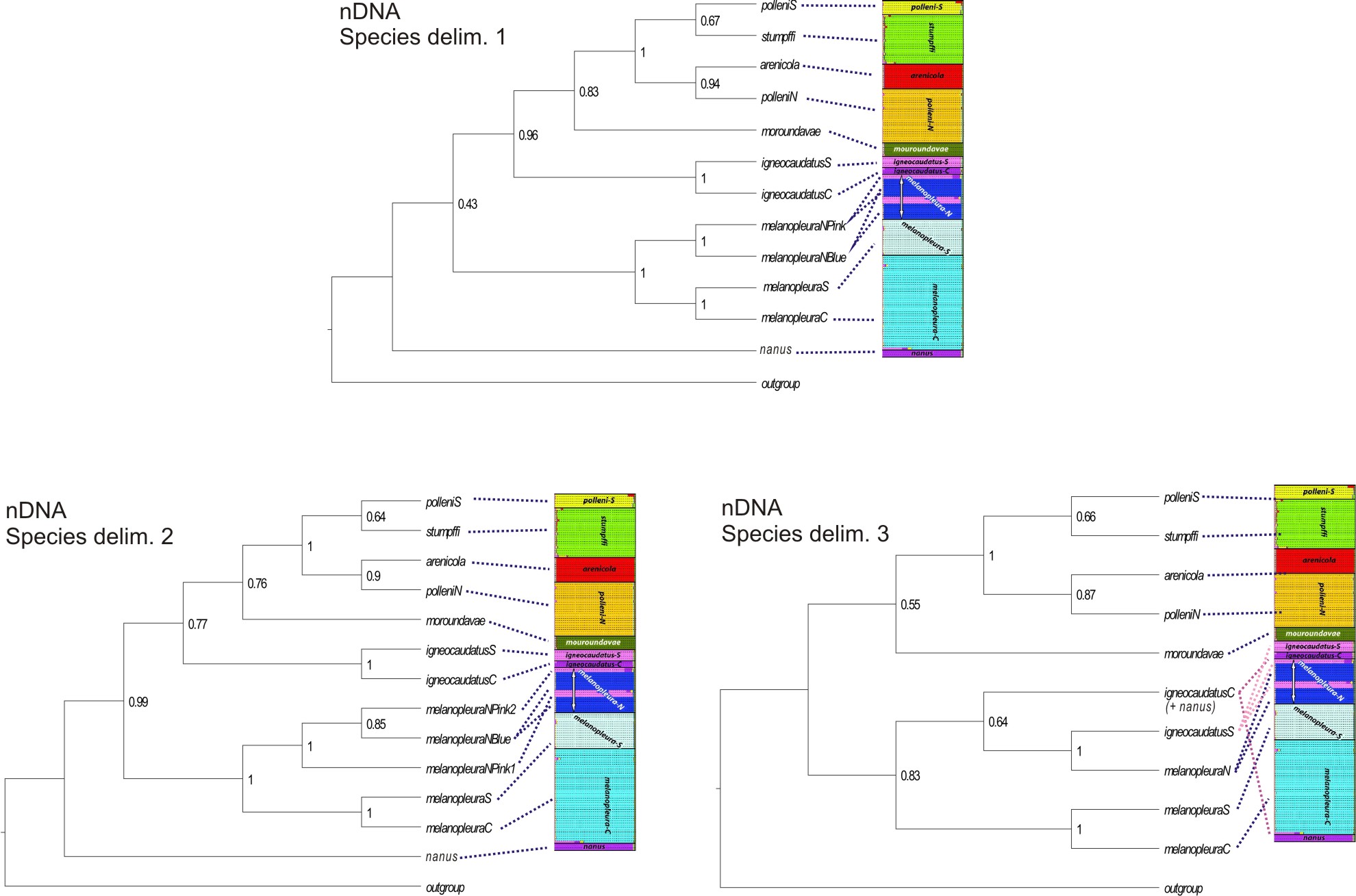


**References :**

Drummond AJ, Suchard MA, Xie D, Rambaut A (2012) Bayesian phylogenetics with BEAUti and the BEAST 1.7. Mol Biol Evol. 1969-1973.

Li B, Lecointre G (2009). Formalizing reliability in the taxonomic congruence approach. Zool. Scripta. 38:101–112.

Liu L, Yu L, Kubatko L, Pearl DK, Edwards SV (2009) Coalescent methods for estimating phylogenetic trees. Mol Phylogenet Evol. 53:320-328.
