# Supplementary material for: New Metrics for Comparison of Taxonomies Reveal Striking Discrepancies among Species Delimitation Methods in Madascincus Lizards
Source: PLoS One. 2013 Jul 12;8(7):e68242. doi: 10.1371/journal.pone.0068242 (PMC3710018; doi:10.1371/journal.pone.0068242)
Supplement: File S7 — Bayesian Assignment tests: complementary analyses. a. STRUCTURE analyses based on complete dataset (all taxa) and data subsets, with subsequent solutions of higher K. b. Taxonomic congruence (Ctax) of the corrected and uncorrected BAT results. (DOC) [file pone.0068242.s007.doc]

**S7. Bayesian Assignment tests: complementary analyses**

**S7a.** STRUCTURE analyses based on complete dataset (all taxa) and data subsets, with subsequent solutions of higher K. In addition to the analyses based on the complete set of taxa (a), complementary analyses have been repeated for subsets of taxa, i.e., separately for the *polleni-stumpffi-arenicola* clade (b) and the *melanopleura* clade (c) which both comprise morphologically similar taxa and were highly supported as monophyletic groups in all analyses. Clusters obtained in the STRUCTURE plot (based on nuclear DNA only) are shown with their correspondence to clades in the mtDNA gene Bayesian Inference (BI) tree.Analyses were performed under models assuming a range of 2 to 18 populations (K) for the complete set of taxa, of 2 to 6 for the *polleni-stumpffi-arenicola* clade and of 2 to 7 for the *melanopleura* clade. (see details in Material and Methods section). The optimal clustering solution retained is K=10 for the complete set of taxa, K=4 for the *polleni-stumpffi-arenicola* clade and K=3 for the *melanopleura* clade. (cf. Material and Methods section for details).

For each analysis, optimal clustering and other subsequent solutions of the higher K values are shown and compared with mtDNA BI tree. For the *polleni-stumpffi-arenicola* clade, complete (a) and separated (b) analyses give identical results, supporting K=4 populations, congruently with the mtDNA tree topology. For the *melanopleura* clade, the separated analysis (c) supports the existence of K=3 populations, consistent with the mtDNA tree topology and fitting perfectly the geographic distribution of each cluster. On the contrary, the complete analysis (a) supports 4 populations, some individual of the *melanopleura*-N clade clustering with the *igneocaudatus*-S clade. This result is herein interpreted as an artifact given its high incongruence with the mtDNA tree, the high morphological divergence observed between these two taxa, and their distinct distribution ranges (see Results section).

| 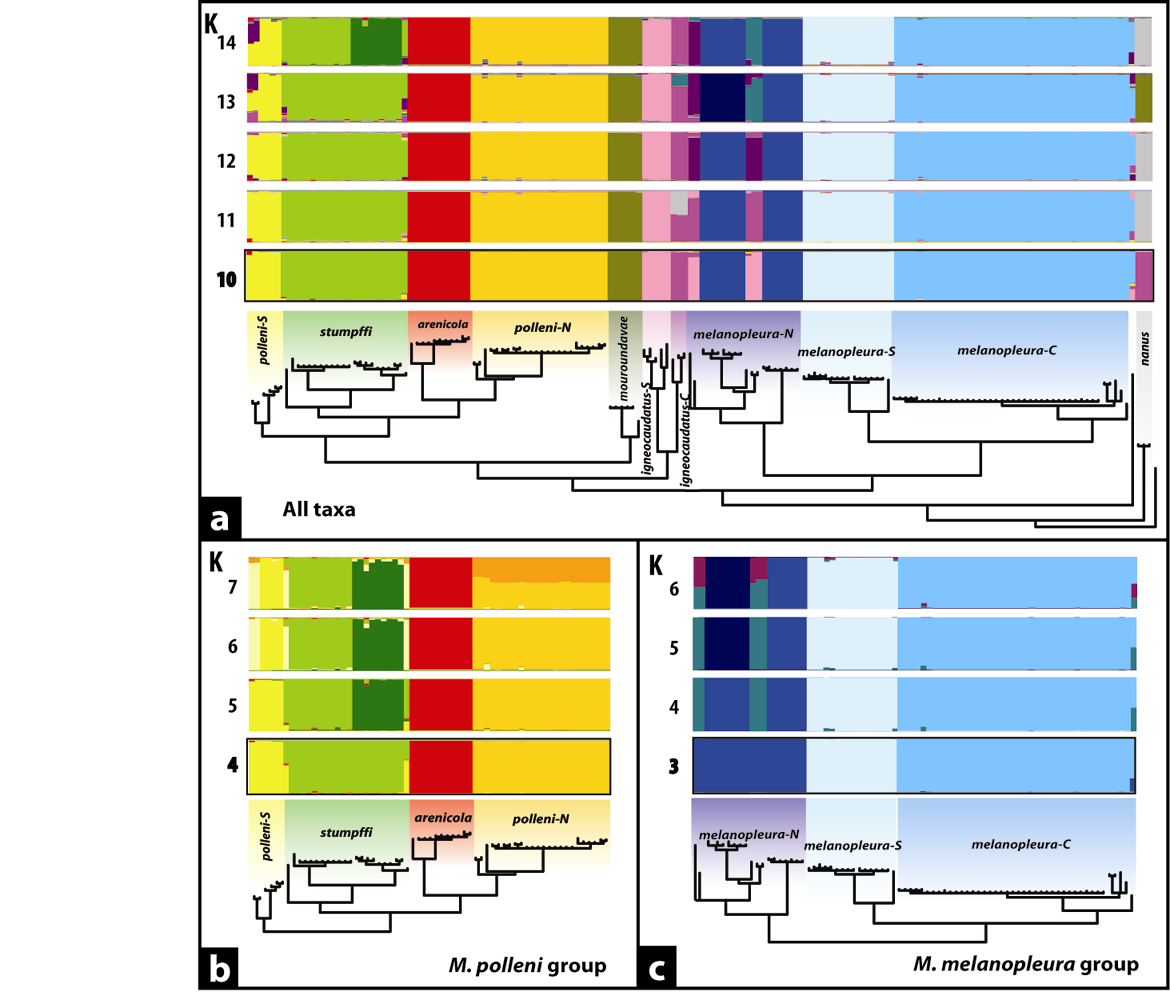 |
| --- |

**S7b.** Taxonomic congruence (Ctax) of the corrected and uncorrected BAT results.The corrected BAT (using the STRUCTURE analyses of taxon subsets to correct the “artifacts” identified in the analysis based on the complete set of individuals) appears to be always more congruent with all the other methods than the uncorrected BAT (results directly inferred from the assignment obtained by the complete analysis, without correcting the probable artifacts). It is important to notice that calculating Ctax using the uncorrected BAT assumes that nuclear clusters of specimens suggested by the complete STRUCTURE analysis represent monophyletic groups (monophyly being a prerequisite of the Ctax algorithm), implying that the mtDNA gene tree would not reflect the true species tree topology (e.g., due to introgression).

|  | **BAT** | **Uncorrected BAT** |
| --- | --- | --- |
| **BAT** | N/A | - |
| **Uncorrected BAT** | 0.70 | N/A |
| **MTMC** | 0.80 | 0.50 |
| **HW** | 0.57 | 0.50 |
| **BSD** | 0.53 | 0.47 |
| **GMYC** | 0.30 | 0.27 |
| **ITAX** | 0.91 | 0.64 |
| **WP** | 0.57 | 0.43 |
