# Supplementary material for: New Metrics for Comparison of Taxonomies Reveal Striking Discrepancies among Species Delimitation Methods in Madascincus Lizards
Source: PLoS One. 2013 Jul 12;8(7):e68242. doi: 10.1371/journal.pone.0068242 (PMC3710018; doi:10.1371/journal.pone.0068242)
Supplement: File S8 — Workflow and application of the ITAX protocol. (DOC) [file pone.0068242.s008.doc]

**S8. Workflow and application of the ITAX protocol.**

A total of four criteria strongly supporting species boundaries were applicable to the complete *Madascincus* dataset (mtDNA, nDNA, morphology and complementary eco-ethological information):

**Evidence from sympatric occurrence without admixture :**

(i) evidence for at least one fixed and unambiguous phenotypic character difference (i.g. presence or absence for qualitative characters, non-overlapping values for meristic or mensural characters, pattern of coloration unambiguously differentiated, or distinct modes of reproduction) between two sister mtDNA lineages know to occur in some instance in sympatry.

(ii) evidence for absence of gene flow (distinct fields of haplotype recombination sensu Flot et al. (2010)) from at least one nuclear gene between two sister mtDNA lineages known to occur in some instance in sympatry.

**Correlation of multiple lines of evidence (sympatry not required):**

(iii) evidence for absence of gene flow (distinct fields of haplotype recombination sensu Flot et al. (2010) for most of the nuclear genes (at least three out of four) between sister mtDNA lineages.

(iv) correlated evidence for divergence in at least two different sets of characters (morphological, eco-ethological or nuclear) between two sister mtDNA lineages which are not necessarily sympatric.

Additional criteria (e.g. strong differences in a behavioural, morphological or genetic characters known to mediate premating isolation, or unviability or unfertility of hybrids) were not applicable to the present dataset. The aim of the ITAX approach being to minimize the alpha error, we considered that five individuals per species represent the minimal acceptable sampling to reliably support the distinctiveness of a given species. Thus, species revealed by this protocol represented by an insufficient sampling were not validated and were consequently merged with their sister species. Two taxa (*M. nanus* and *igneocaudatus-C*, each represented by only three DNA samples) were nevertheless regarded as exceptions to this rule given that (1) more than five collection specimens were examined for morphology and (2) their distinctiveness was strongly supported by multiple evidence of absence of gene flow and by very obvious eco-ethological and morphological divergences.

**Reference:**

Flot J-F, Couloux A, Tillier S (2010) Haplowebs as a graphical tool for delimiting species: a revival of Doyle’s “field for recombination” approach and its application to the coral genus *Pocillopora* in Clipperton. BMC Evol. Biol. 10:372.
