# Supplementary material for: New Metrics for Comparison of Taxonomies Reveal Striking Discrepancies among Species Delimitation Methods in Madascincus Lizards
Source: PLoS One. 2013 Jul 12;8(7):e68242. doi: 10.1371/journal.pone.0068242 (PMC3710018; doi:10.1371/journal.pone.0068242)
Supplement: File S9 — Workflow and application of the WP protocol. (DOC) [file pone.0068242.s009.doc]

**S9. Workflow and application of the WP protocol**

This protocol follows the methodology published by Wiens and Penkrot (2002) to delimit species using a DNA haplotype phylogeny, and a schematic example of the flow chart proposed by these authors has been redrawn below. Focal species were recognized as those nominal species listed in the taxonomic framework section (*cf.* Material and Methods). Taxonomic status was tested for each of them independently, using the BI mtDNA tree topology. Following recommendations formulated by Wiens and Penkrot, a number of two individuals was considered as the minimal sampling acceptable to support the distinctiveness of a given species. Thus, species revealed by this protocol that were represented by a single haplotype were not validated and were consequently merged with their sister species. Nevertheless, in the rare cases where this distinct single haplotype is sister species of a clade already composed by several species supported by this approach (themselves represented by two or more samples), we exceptionally considered this single haplotype as an additional distinct species, to be in accordance with the concept of species as monophyletic lineages in the gene genealogy, on which this protocol is based. Although this method emphasizes the basal lineages of a focal species as potentially distinct species, it is theoretically possible that each of these basal lineages might contain multiple species (which could be exclusive or nonexclusive). Therefore, the same rationale outlined above to detect such cases was repeated until reaching stability of the species boundaries inferred (Wiens and Penkrot, 2002). The hypothetical example (redrawn from Wiens and Penkrot (2002)) presented below involves two species, one with two disjunct populations (species A), the other with a single contiguous population (species B). Two individuals are sampled from each population (e.g., A1 = a haplotype from locality 1 of species A). The horizontal bars above the terminal taxa indicates species limits.


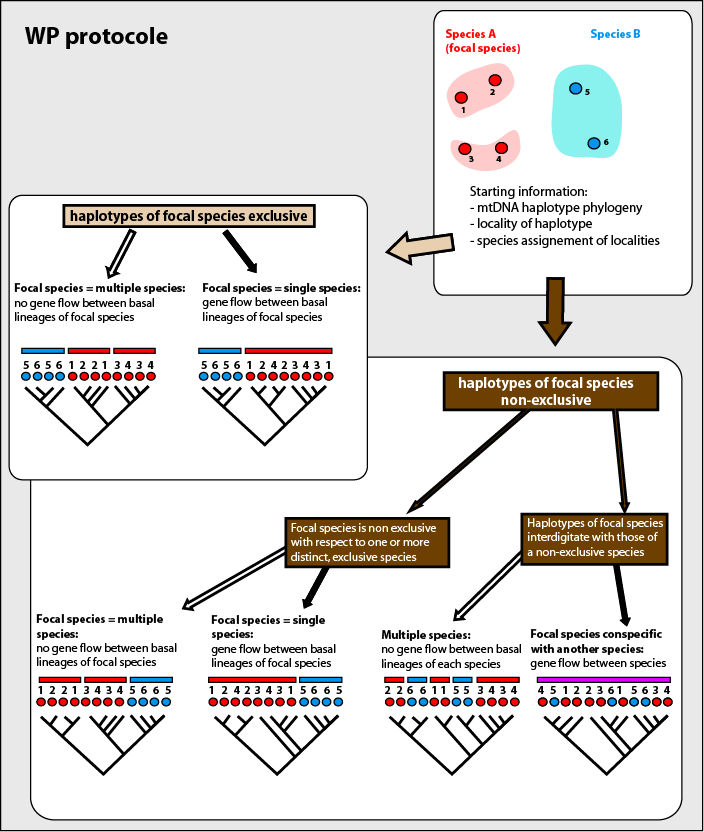


**Reference :**

Wiens JJ, Penkrot TA (2002). Delimiting species using DNA and morphological variation and discordant species limits in spiny lizards (*Sceloporus*). Syst. Biol. 51:69–91.
