# Supplementary material for: New Metrics for Comparison of Taxonomies Reveal Striking Discrepancies among Species Delimitation Methods in Madascincus Lizards
Source: PLoS One. 2013 Jul 12;8(7):e68242. doi: 10.1371/journal.pone.0068242 (PMC3710018; doi:10.1371/journal.pone.0068242)
Supplement: File S10 — Partitioning and combination of priors for Bayesian Species Delimitation (BSD) analysis. (DOC) [file pone.0068242.s010.doc]

**S10. Partitioning and combination of priors for Bayesian Species Delimitation (BSD) analysis**

**Prior:** The prior distributions of the ancestral population size (θ) and root age (τ_o_) can affect the posterior probability of models, with large values for θ and small values for τ_o_ favoring conservative models containing fewer species (Yang & Rannala 2010). We evaluated the influence of these priors by considering three different combinations as in Leaché and Fujita (2010), assigning both priors a gamma G(α, β) distribution, with a prior mean = α/β and prior variance = α/β^2^ The first combination assumed relatively large ancestral population sizes and deep divergences: θ ~ G(1, 10) and τ_o_ ~ G(1, 10), both with a prior mean = 0.1 and variance = 0.01. The second combination of priors assumed relatively small ancestral population size and shallow divergences among species: θ ~ G(2, 2000) and τ_o_ ~ G(2, 2000), both with a prior mean = 0.001 and variance = 5x10^-7^. The final combination is a mixture of priors that assume large ancestral population size θ ~ G(1, 10) and relatively shallow divergence among species τ_o_ ~ G(2, 2000), which is a conservative combination of priors that should favor models containing fewer species (Leaché and Fujita, 2010)).

**Partitioning and guide tree:** For practical reasons, analyses have been applied separately on four different species groups of *Madascincus* which unambiguously constitute distinct heterospecific clades: the *M. polleni* group, the *M. melanopleura* group, the *M. igneocaudatus* group and the *M. mouroundavae* group. This division was applied to reduce the running time of the calculations to a realistic level, and to reduce the reliance of the analysis on some deep nodes in the tree that could not be resolved with full reliability due to conflicts among partitioning schemes or data sets. For each of the groups, ten, nine, six and two lineages have been considered as potential candidate species. Content of each of these candidate species and user-specified guide tree (based on mtDNA BI) have been implemented in BBP as following:

| ***Analysis of the M. polleni group* (ten candidate species):**  **guide tree =** **((((AR-a, AR-b),((PN-c, PN-b), PN-a)), ((ST-c, ST-b), ST-a)), (PS-a, PS-b))** | |
| --- | --- |
| *M. arenicola* : | AR-a (n=1, Ampobofofo)  AR-b (n=10, Antsiranana, Orangea). |
| *M. polleni* southern clade : | PS-a (n=2, Ankarafantsika)  PS-b (n=4, Kirindy). |
| *M. polleni* northern clade : | PN-a (n=2, Ankarana)  PN-b (n=1, Ampobofofo)  PN-c (n=21, Montagne des Français, Orangea). |
| *M. stumpffi* : | ST-a (n=1, Antanambao);  ST-b (n=11, Marojejy);  ST-c (n=10, Antsirasira, Montagne des Français, Montagne d’Ambre) |
| ***Analysis of the M. melanopleura group* (nine candidate species):**  **guide tree = (((MS-a, MS-b)(MC-a, MC-b))(MN-a (MN-e (MN-c (MN-d, MN-b)))))** | |
| central clade: | MC-a (n=1, Ambohitsara)  MC-b (n=41, Anala2, Andasibe, Fierenana, Mahasoa, Sahafina, Torotorofotsy). |
| northern clade: | MN-a (n=7, Nosy Mangabé)  MN-b (n=8, Angozongahy, Makira)  MN-c (n=2, Montagne d’Ambre)  MN-d (n=1, Tsaratanana2)  MN-e (n=2, Anala1, Tsaratana1). |
| southern clade: | MS-a (n=15, Ambatolahy, Ranomafana, Ranomafanakely, Imaloka)  MS-b (n=1, Andohahela) |
| ***Analysis of the M. igneocaudatus* (six candidate species):**  **guide tree: ((IC-a, IC-b), ((IS-a, IS-d), (IS-c, IS-b)))** | |
| central clade: | IC-a (n=1, Itremo);  IC-b (n=2, Ibity). |
| southern clade: | IS-a (n=2, Sakabera, Ifaty)  IS-b (n=1, Anakao)  IS-c (n=1, Faux Cap)  IS-d (n=1, Ifaty) |
| ***Analysis of the M. mouroundavae* (two candidate species):**  **guide tree = (MOa, MOb)** | |
| *M. mouroundavae :* | MO-a (n=5, Andasibé)  MO-b (n=1, Montagne d’Ambre) |

**References :**

Yang Z, Rannala B (2010) Bayesian species delimitation using multilocus sequence data. Proc. Nat. Acad. Sci. USA. 107:9264–9269.

Leaché, AD. Fujita, MK (2010) Bayesian species delimitation in West African forest geckos (*Hemidactylus fasciatus*). Proc. R. Soc. B. 278:490–492.
