# Supplementary material for: New Metrics for Comparison of Taxonomies Reveal Striking Discrepancies among Species Delimitation Methods in Madascincus Lizards
Source: PLoS One. 2013 Jul 12;8(7):e68242. doi: 10.1371/journal.pone.0068242 (PMC3710018; doi:10.1371/journal.pone.0068242)

**S11. Haploweb reconstructions for the four nuclear genes (BDNF, PDC, CMOS and RAG2)**

For each marker, circles represent haplotypes (size proportional to the number of individuals), black lines represent mutational steps and black dots missing haplotypes, white curves represent connections between haplotypes found co-occurring in heterozygous individuals, and white numbers represent the number of individuals in which the respective haplotypes were found co-occurring. Single locus fields of recombination (pools of co-occurring haplotypes) are represented by grey rectangles. Note that only haplotype sharing and not the connections between haplotypes are taken into account for species delimitation.


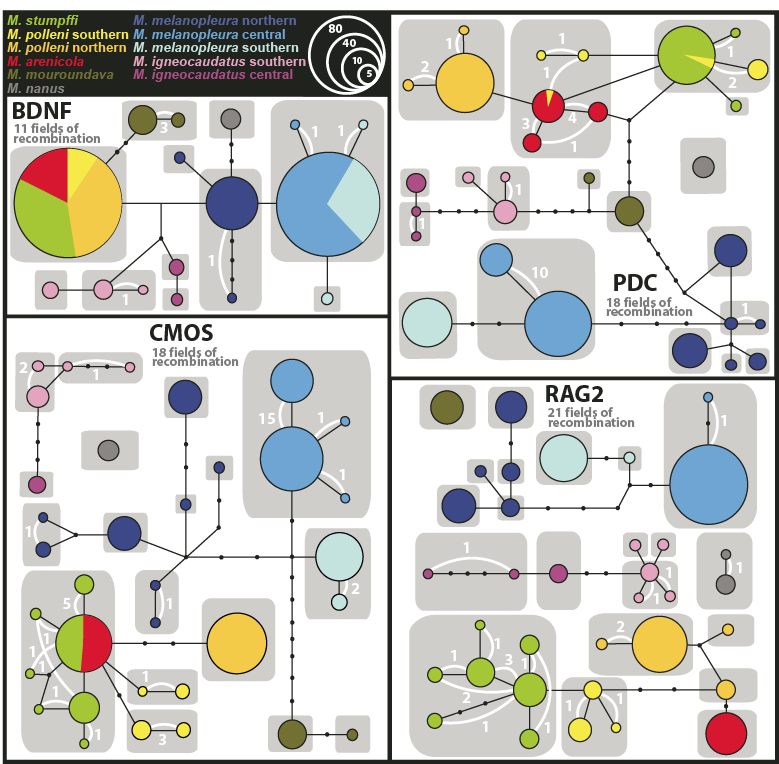

Supplement: File S11 — Haploweb reconstructions for the four nuclear genes (BDNF, PDC, CMOS and RAG2). (DOC) [file pone.0068242.s011.doc]
